# Unmasking true confinement effects: ultrahigh linear selectivity and chain-length oscillatory behavior in zeolite-encapsulated rhodium hydroformylation

**DOI:** 10.1093/nsr/nwag110

**Published:** 2026-02-23

**Authors:** Tao Yan, Xiangjie Zhang, Gengzhe Song, Ziyu Zhou, Huaming Hou, Qingqing Xie, Huizi He, Xin Tong, Hongying Chang, Zhiqiang Liu, Anmin Zheng, Zhi Cao, Peng He

**Affiliations:** State Key Laboratory of Coal Conversion, Institute of Coal Chemistry, Chinese Academy of Sciences, Taiyuan 030001, China; National Energy Center for Coal to Clean Fuels, Synfuels China Technology Co., Ltd., Beijing 101407, China; School of Chemical Sciences, University of Chinese Academy of Sciences, Beijing 100190, China; National Energy Center for Coal to Clean Fuels, Synfuels China Technology Co., Ltd., Beijing 101407, China; National Energy Center for Coal to Clean Fuels, Synfuels China Technology Co., Ltd., Beijing 101407, China; National Energy Center for Coal to Clean Fuels, Synfuels China Technology Co., Ltd., Beijing 101407, China; National Energy Center for Coal to Clean Fuels, Synfuels China Technology Co., Ltd., Beijing 101407, China; State Key Laboratory of Coal Conversion, Institute of Coal Chemistry, Chinese Academy of Sciences, Taiyuan 030001, China; National Energy Center for Coal to Clean Fuels, Synfuels China Technology Co., Ltd., Beijing 101407, China; School of Chemical Sciences, University of Chinese Academy of Sciences, Beijing 100190, China; State Key Laboratory of Coal Conversion, Institute of Coal Chemistry, Chinese Academy of Sciences, Taiyuan 030001, China; National Energy Center for Coal to Clean Fuels, Synfuels China Technology Co., Ltd., Beijing 101407, China; School of Chemical Sciences, University of Chinese Academy of Sciences, Beijing 100190, China; State Key Laboratory of Coal Conversion, Institute of Coal Chemistry, Chinese Academy of Sciences, Taiyuan 030001, China; National Energy Center for Coal to Clean Fuels, Synfuels China Technology Co., Ltd., Beijing 101407, China; School of Chemical Sciences, University of Chinese Academy of Sciences, Beijing 100190, China; School of Chemical Sciences, University of Chinese Academy of Sciences, Beijing 100190, China; Interdisciplinary Institute of NMR and Molecular Sciences, Key Laboratory of Hubei Province for Coal Conversion and New Carbon Materials, School of Chemistry and Chemical Engineering, Wuhan University of Science and Technology, Wuhan 430081, China; Interdisciplinary Institute of NMR and Molecular Sciences, Key Laboratory of Hubei Province for Coal Conversion and New Carbon Materials, School of Chemistry and Chemical Engineering, Wuhan University of Science and Technology, Wuhan 430081, China; State Key Laboratory of Coal Conversion, Institute of Coal Chemistry, Chinese Academy of Sciences, Taiyuan 030001, China; National Energy Center for Coal to Clean Fuels, Synfuels China Technology Co., Ltd., Beijing 101407, China; School of Chemical Sciences, University of Chinese Academy of Sciences, Beijing 100190, China; State Key Laboratory of Coal Conversion, Institute of Coal Chemistry, Chinese Academy of Sciences, Taiyuan 030001, China; National Energy Center for Coal to Clean Fuels, Synfuels China Technology Co., Ltd., Beijing 101407, China; School of Chemical Sciences, University of Chinese Academy of Sciences, Beijing 100190, China

**Keywords:** Rh-catalyzed hydroformylation, selective passivation, zeolite confinement, resonant diffusion, regioselectivity

## Abstract

Hydroformylation of higher *α*-olefins with high linear selectivity is hampered by rapid isomerization and lack of steric control in conventional heterogeneous catalysts. Here, we selectively passivate external rhodium (Rh) sites on a zeolite-encapsulated Rh@MEL catalyst using bulky 2,4-dimethylbenzenethiol (DMBT), producing Rh@MEL-DMBT that exposes only micropore-confined single Rh sites within 5.3 Å × 5.4 Å MEL channels. This eliminates interference from unrestricted surface Rh, enabling unambiguous probing of true shape-selective hydroformylation. Rh@MEL-DMBT delivers exceptional performance for C_5_–C_12_  *α*-olefins, achieving linear-to-branched (*l*/*b*) ratios up to 600, >95% aldehyde chemoselectivity, and significant suppression of isomerization (<5% internal olefins) observed for most substrates within this range. Strikingly, activity and aldehyde selectivity exhibit sinusoidal oscillation with increasing chain length which is directly linked to resonant diffusion of olefins in toluene-filled pores revealed by molecular dynamics simulations. *In situ* Fourier-transform infrared spectroscopy confirms encaged [HRh(CO)_2_] and [HRh(CO)] intermediates, while kinetic isotope effect (KIE = 1.2) and pressure-dependent kinetics rigorously identify CO insertion as the rate-determining step. These results establish the first experimentally validated molecular mechanism of zeolite-confined single-site Rh hydroformylation and unveil diffusion-controlled chain-length-dependent regioselectivity in confined catalysis.

## Teaser

Selective passivation of the external Rh in Rh@MEL achieves highly regioselective hydroformylation toward linear aldehydes and reveals a sinusoidal pattern with increasing substrate chain length.

## INTRODUCTION

Hydroformylation—the catalytic addition of syngas (CO/H_2_) to olefins to produce aldehydes—remains a cornerstone reaction for olefin valorization and one of the largest homogeneous catalytic processes worldwide, with an annual production capacity exceeding 10 million tons [1,2]. Despite its maturity, the solubility of conventional rhodium catalysts severely complicates separation and recycling, resulting in significant metal loss and raising both economic and environmental concerns [3]. Immobilizing Rh as a reusable heterogeneous catalyst offers an attractive solution, yet classical supported systems lack the sophisticated ligand environment of their homogeneous counterparts [4–7]. Consequently, the archetypal ‘HRh(CO)_2_’ active species operates without steric guidance, delivering poor regioselectivity toward the industrially preferred linear aldehyde. Our group recently reported a breakthrough heterogeneous Rh-zeolite platform that exploits the shape-selective confinement of zeolite micropores. By precisely encapsulating [Rh(CO)_2_]+ sites within narrow channels, the formation of bulky branched intermediates is effectively suppressed in propene hydroformylation, achieving >99% regioselectivity to *n*-butyraldehyde, >99% chemoselectivity to aldehydes, and turnover frequencies exceeding 6500 h^−^^1^—performance that surpasses nearly all known systems and redefines the potential of shape-selective hydroformylation [8].

**Figure 1.**
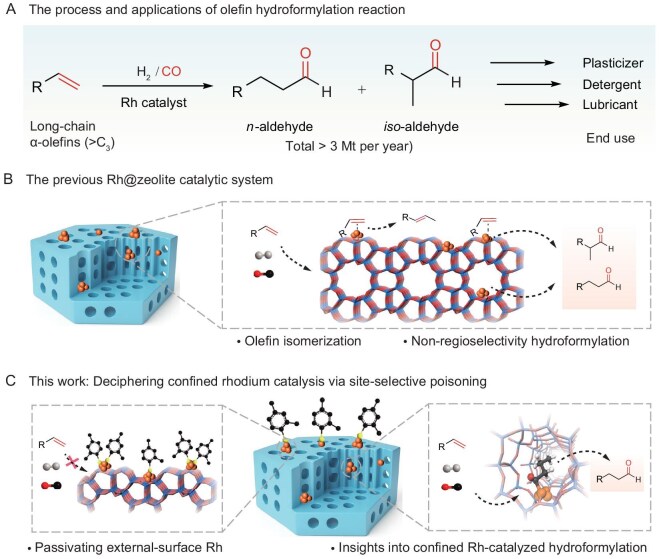
Background of the hydroformylation of long-chain olefins and our proposed catalytic system. (A) Hydroformylation reaction process and applications. (B) Rhodium species are present on both the internal and external surfaces of the Rh@zeolite catalyst. Multiple parallel reaction pathways occur on the unconfined external Rh sites, leading to non-selective product formation. (C) Our strategy of selectively passivating the external surface Rh sites to probe the authentic reaction pathway of zeolite-confined Rh sites. The black, white, yellow, red, and orange balls represent C, H, S, O, and Rh atoms, respectively.

For higher α-olefins (>C3), obtaining high regioselectivity to long-chain linear aldehydes is of paramount industrial importance, as these products serve as key precursors to linear alcohols and carboxylic acids used in plastics, detergents, and lubricants [9,10] (Fig. 1A). Unlike propylene, longer α-olefins suffer from rapid competitive isomerization, in which the terminal double bond migrates to a thermodynamically favored internal position. Once formed, these internal olefins undergo hydroformylation to yield branched aldehydes, dramatically broadening the product distribution and complicating the reaction network [11,12]. Rh-zeolite catalysts elegantly circumvent this challenge by exploiting spatial constraints within the micropores, delivering significantly enhanced linear-aldehyde selectivity even for higher substrates [13–16]. Nevertheless, a persistent limitation remains: irrespective of the preparation method—impregnation or direct encapsulation—rhodium clusters inevitably form on the external zeolite surface [17–19]. At these unrestricted sites, the coordinated olefin exhibits little preference for linear versus branched insertion, producing roughly equimolar mixtures of linear and branched aldehydes. In stark contrast, rhodium species confined within the micropores benefit from severe steric hindrance that disfavors bulky branched transition states, thereby affording near-perfect linear selectivity.

Deciphering the intrinsic catalytic mechanism of truly encaged rhodium in hydroformylation has therefore proven extraordinarily challenging [20]. Previous mechanistic investigations have invariably been confounded by the coexistence of intra- and extra-framework rhodium species, which operate through fundamentally different pathways yet have rarely been distinguished [13,21] (Fig. 1B). As a result, the genuine role of pore-confined rhodium has remained obscured. Moreover, the encapsulated metal lies deeply buried within the zeolite lattice, with only a portion of Rh atoms at the metal–zeolite interface exposed to the reaction medium—and an even smaller subset capable of participating in catalysis [22]. Identifying the true active centers within these complex, spatially restricted environments is thus very difficult. This inherent complexity has frequently led to mechanistic conclusions being drawn from oversimplified or misleading catalyst models, yielding structure–activity relationships that deviate substantially from reality.

By selectively passivating external-surface rhodium, the present work aims to isolate the contribution of micropore-confined sites, thereby unveiling the authentic performance of encaged rhodium and providing molecular-level insight into shape-selective hydroformylation catalysis (Fig. 1C).

## RESULTS AND DISCUSSION

### Synthesis and characterization of Rh@MEL and Rh@MEL-DMBT catalysts

**Figure 2.**
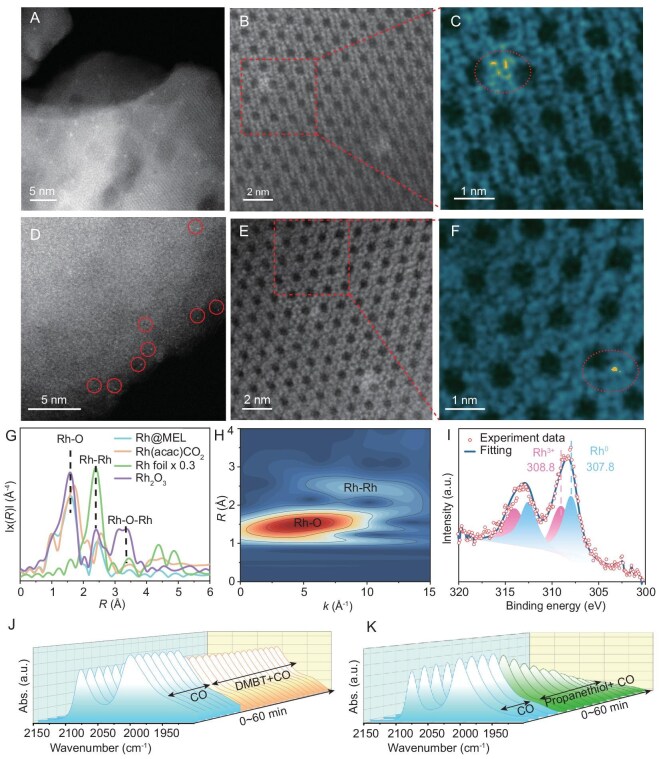
Comprehensive characterization of the Rh@MEL and Rh@MEL-DMBT. (A–F) Aberration-corrected HAADF-STEM images of Rh@MEL. Isolated Rh atoms in the sample are marked by circles in panel (D). (C and F) Colored HAADF-STEM image of the area outlined in (B) and (E), respectively. (G) Fourier transform EXAFS spectra with *k*^3^-weighted magnitude at the Rh K-edge of different Rh catalysts and reference samples. (H) Wavelet-transformed EXAFS spectra at the Rh K-edge. (I) Rh 3d XPS spectra of Ar^+^-sputtered Rh@MEL catalysts. (J and K) Time evolution of CO stretching vibration intensity of the [Rh(CO)_2_] species. The Rh@MEL catalyst was exposed to a CO atmosphere containing either DMBT (J) or propanethiol (K).

Using a modified one-pot hydrothermal crystallization method [8,23], rhodium species were successfully incorporated into a siliceous MEL-type zeolite framework, designated as Rh@MEL. The Rh loading was determined by inductively coupled plasma optical emission spectroscopy (ICP-OES) to be 0.32 wt% (Table S1). The spatial distribution of Rh species in MEL zeolite (pore size: 5.3 × 5.4 Å) was probed by contrasting the hydrogenation rates of 1-octene (kinetic diameter: 5.3 Å) and cyclooctene (kinetic diameter: 8.0 Å) over Rh@MEL and Rh/SiO_2_, following reported methods [24,25]. This assessment revealed that the majority of Rh is encapsulated within the framework, while ∼6% of Rh species remain accessible on the external surface (Table S2). X-ray diffraction (XRD) patterns confirmed that the crystalline structure of the zeolite remained intact, with no detectable diffraction peaks corresponding to crystalline Rh or Rh_2_O_3_ (Fig. S1), indicating the absence of large Rh-containing aggregates. The N_2_ adsorption-desorption isotherms indicate that the channels are not significantly obstructed by the Rh species (Fig. S2). A rugby-ball-like morphology and uniform particle size of the zeolite samples were displayed by scanning electron microscopy (SEM) images (Fig. S3). The local structure of Rh was examined by aberration-corrected high-angle annular dark-field scanning transmission electron microscopy (HAADF-STEM), which revealed the coexistence of subnanometer Rh clusters (Fig. 2A) and isolated single atoms (Fig. 2D) within the zeolite framework (Figs S4 and S5). The spatial distribution of Rh species within the MEL zeolite was revealed by HAADF-STEM imaging along the <100> direction (Fig. 2B and E). Smaller Rh clusters and isolated single Rh atoms are located around the 5- and 6-membered rings (Fig. 2C and F). Similar localization has been observed for Pt species within the MFI framework [26]. Elemental mapping further demonstrated a homogeneous distribution of Rh throughout the zeolite matrix (Fig. S6). Structural information of the Rh species in Rh@MEL was provided by CO probe Fourier-transform infrared (FTIR) spectroscopy. The IR spectrum exhibited two bands at 2084 and 2007 cm^−1^ (Fig. S7), which are attributed to the symmetric and asymmetric CO stretching modes of gem-dicarbonyl [Rh(CO)_2_] species [27,28]. The IR spectral feature demonstrates high dispersion of Rh and a relatively uniform active site structure within the MEL framework, which is consistent with previous studies [8].

The local coordination environment and electronic state of the Rh species were further investigated using X-ray absorption spectroscopy. Fourier-transform analysis of the extended X-ray absorption fine structure (EXAFS) spectra (Fig. 2G and Fig. S8) indicated that the first coordination shell of Rh in Rh@MEL is dominated by Rh–O scattering paths, with a much weaker Rh–Rh contribution. The absence of a second-shell Rh–O–Rh scattering path rules out the formation of Rh_2_O_3_-like nanoparticles, which is consistent with high dispersion of Rh. This finding was further corroborated by wavelet transform (WT) EXAFS analysis (*k* = 3–15 Å^−1^, Morlet wavelet), where the 2D contour plots show no detectable intensity corresponding to Rh–O–Rh contributions for the Rh@MEL sample (Fig. 2H). Further analysis revealed that each Rh atom is coordinated with ∼3.6 oxygen atoms in the first coordination shell (Table S3). The Si–OH groups or defect sites within the zeolite framework, as corroborated by IR and nuclear magnetic resonance (NMR) spectroscopy (Figs S9 and S10), stabilize Rh single atoms and clusters via the formation of Rh_x_–O_y_–Si species [17,29], resulting in a higher abundance of Rh–O coordination. The X-ray absorption near-edge structure (XANES) spectra of Rh@MEL closely resemble those of the [Rh(acac) (CO)_2_] reference compound, indicating that the Rh species are predominantly in a partially oxidized state (Fig. S11). X-ray photoelectron spectroscopy (XPS) was employed to further evaluate the electronic state of Rh. To access these encapsulated species, Ar^+^ sputtering was systematically applied to remove surface layers while maintaining structural integrity (Fig. 2I). Subsequent XPS analysis in the Rh 3d region clearly reveals mixed valence states, with characteristic binding energies observed at 308.8 eV (Rh^3+^, 3d_5/2_) and 307.8 eV (Rh^0^, 3d_5/2_). The charge polarization state of the Rh species is consistent with the XANES results. These characteristics of Rh@MEL collectively confirm the dominance of strongly anchored, highly dispersed Rh species interacting with the zeolite framework.

To investigate the authentic performance of confined rhodium species, it is essential to selectively passivate external-surface rhodium and thus isolate the contribution of micropore-confined sites. Selective poisoning with DMBT was performed to passivate the Rh sites on the external surface of Rh@MEL, since its molecular size (kinetic diameter: 8.1 Å) is larger than the pore size of the MEL zeolite (pore size: 5.3 Å × 5.4 Å) and the sulfur atom could then bond to the Rh atoms. The resulting catalyst poisoned with DMBT was termed Rh@MEL-DMBT. To validate our hypothesis, an *in situ* IR study was performed to monitor the adsorption of DMBT on the Rh species. The IR spectra of Rh@MEL under a CO environment revealed typical geminal dicarbonyl [Rh(CO)_2_] signals at 2084 and 2007 cm^−1^. Upon gradual addition of DMBT in the CO flow, the intensity of the CO bands decreased slightly and rapidly stabilized (Fig. 2J), suggesting that DMBT could form a stronger bond with Rh than CO molecules, enabling DMBT to compete with CO and poison Rh sites during the hydroformylation reaction. It is noteworthy that the reduced absorptance of the IR peak is estimated to be ∼10%, similar with the proportion of external Rh sites in Rh@MEL (6%), indicating that DMBT selectively adsorbs on the external Rh sites and cannot access the Rh sites located inside MEL pores. We also selected propanethiol, which has a smaller kinetic diameter (4.6 Å) and can diffuse into the pore channels to access all Rh sites, as a reference molecule. Unlike DMBT, when propanethiol was introduced (Fig. 2K), the intensity of the CO vibration bands continuously decreased, suggesting adsorption of propanethiol on intra- and extra-framework Rh sites. These IR experiments demonstrate that the Rh sites can be effectively poisoned by thiols under reaction conditions. Moreover, the bulky DMBT enables selective poisoning of the external Rh sites, without affecting the Rh sites within the zeolite framework. Therefore, the Rh@MEL-DMBT catalyst allows us to directly probe the reaction pathways associated exclusively with the confined Rh sites within the zeolite framework.

### Catalytic performance for long-chain *α*-olefins hydroformylation by Rh@MEL-DMBT

The hydroformylation of propylene is immune to alkene isomerization due to its three-carbon molecule structure. However, when long-chain *α*-olefins were the hydroformylation feedstocks, their isomerization to internal olefins constitutes a significant side reaction. Since the direct hydroformylation of these internal olefins predominantly yields branched aldehydes, this isomerization pathway inevitably shifts the product distribution toward branched isomers, substantially diminishing the *l*/*b* ratio [11,30]. Given this inherent challenge with long-chain olefins, it becomes crucial to distinguish between the contributions of alkene isomerization and hydroformylation at different Rh sites. Therefore, we selected 1-hexene as a model substrate to probe the catalytic behavior of Rh@MEL-DMBT.

**Figure 3.**
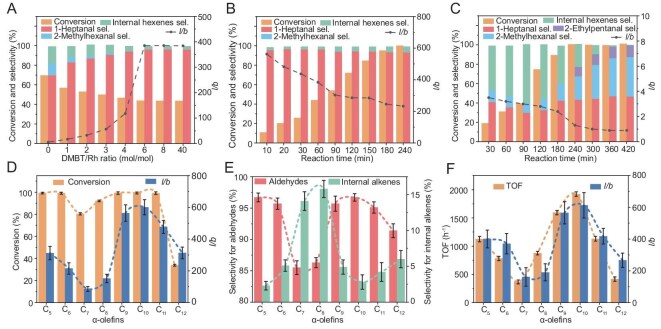
Catalytic performance of Rh@MEL-DMBT in hydroformylation of long-chain *α*-olefins. (A) Optimization of DMBT dosage using 1-hexene as the substrate. Time-dependent catalytic performance of Rh@MEL-DMBT (B) and Rh/SiO_2_ (C) for 1-hexene hydroformylation. (D) The conversion and regioselectivity results for the hydroformylation of different linear *α*-olefins over Rh@MEL-DMBT. (E) The selectivity results for the hydroformylation of different linear *α*-olefins over Rh@MEL-DMBT. (F) Initial hydroformylation rates and regioselectivity of different linear *α*-olefins over Rh@MEL-DMBT at ∼10% conversion.

A series of experiments with varying DMBT/Rh molar ratios were conducted (Fig. 3A). The unpoisoned Rh@MEL catalyst afforded the highest olefin conversion; however, it yielded a low aldehyde *l*/*b* ratio of 5.7 and a 12.3% selectivity toward isomeric olefins. Subsequently, DMBT was introduced into the catalytic system via co-feeding with the alkenes. Introducing DMBT led to a marked suppression of both isomeric olefins and branched aldehydes, accompanied by only a slight decrease in linear aldehyde yield. As the DMBT/Rh ratio was increased from 1 to 6, the formation of branched aldehydes was nearly completely inhibited, resulting in an *l*/*b* ratio exceeding 300 while maintaining the isomeric olefin content below 2%. This systematic suppression of isomerization and branched aldehyde, achieved through the progressive deactivation of external Rh sites, strongly indicates that these undesirable pathways are primarily facilitated by Rh species located on the external surface. It is worth noting that the yield of 1-heptaldehyde remained consistent at DMBT/Rh ratio above 6, and higher DMBT dosage induced no significant changes in product distribution. This observation further confirms that the DMBT molecules only passivate the external Rh sites, while those within the MEL pores remain accessible and catalytically active for the desired linear pathway. Furthermore, even at a higher DMBT/Rh ratio of 40, the hydroformylation performance for 1-hexene remained identical to that at a ratio of 6. These results rule out significant pore blockage by DMBT under our experimental conditions, as such blockage would otherwise impede the diffusion of the reactants or products and lower the efficiency of the reaction. With the DMBT dosage optimized, we systematically studied the reaction temperature and pressure needed to optimize the process (Figs S12 and S13). The results show that the conditions of 80°C and 4 MPa provided the highest aldehyde selectivity along with a high olefin conversion; these were selected as the optimal reaction parameters. Notably, under all tested conditions, the *l*/*b* ratio remained above 60, underscoring the powerful confinement effect of the MEL microenvironment in promoting the linear product.

To gain deeper insight into the confinement effect of the zeolite channels throughout the catalytic process, the time-dependent reaction profile of 1-hexene was monitored over Rh@MEL-DMBT. Rh/SiO_2_, a catalyst possessing exclusively external Rh species, was prepared by the impregnation method as the control group. The results demonstrate that Rh@MEL-DMBT effectively suppresses the isomerization of 1-hexene, maintaining byproducts below 5% throughout the reaction, while achieving exceptional regioselectivity (*l*/*b* > 200) toward the linear aldehyde (Fig. 3B). Given the observation of ∼5% internal olefins at complete olefin conversion, we sought to determine whether these isomers would undergo subsequent conversion to branched aldehydes, thereby impacting the overall product selectivity. Hydroformylation reactions were performed using internal olefins as substrates (Table S4). The internal olefins exhibited exceedingly low conversion over prolonged durations and inefficient formation of branched aldehydes. This suggests that the zeolite-confined Rh sites are inherently unresponsive to internal olefins, thereby accounting for the robust regioselectivity observed with long-chain *α*-olefins. This performance presents a striking contrast to that of the supported Rh/SiO_2_ catalyst. For the Rh/SiO_2_ system (Fig. 3C), the highest *l*/*b* ratio attained was only 3.5. Furthermore, internal olefins constituted the primary initial products within the first 2 hours, reaching a maximum yield of over 40%. Their subsequent hydroformylation preferentially formed branched aldehydes, including ∼10% 2-ethylpentanal, which can only be produced from internal olefins. The formation of these branched aldehydes ultimately drove the overall *l*/*b* ratio below 1. This comparison highlights the crucial role of the zeolite confinement in Rh@MEL-DMBT in promoting linear aldehyde formation while effectively suppressing undesirable isomerization pathways. Furthermore, the Rh@MEL-DMBT catalyst demonstrated good stability over six recycling runs, with the Rh loading of the spent catalyst remaining nearly unchanged compared to the fresh catalyst (Table S1). Additionally, the 1‑hexene conversion was maintained at 53.1% after the sixth cycle, close to the initial conversion of 54.8%. Meanwhile, the aldehyde selectivity consistently exceeded 95%, and the *l*/*b* ratio remained above 250 throughout all cycles (Fig. S14).

Subsequently, the substrate scope was expanded to a series of linear *α*-olefins (C_5_–C_12_). Impressive regioselectivity was observed across all substrates, with the *l*/*b* ratio reaching a remarkable value of up to 600 (Fig. 3D and Tables S5–S9). High conversions were generally maintained, although a significant decrease was noted for C_12_ olefin (38%), presumably due to diffusional limitations of the longer-chain reactant or aldehyde product within MEL [31]. Furthermore, scale-up experiments were conducted for the higher-performing olefins (Table S10). At a 10-fold larger scale, catalytic performance remained largely consistent with that observed in small-scale reactions, demonstrating the robustness and scalability of the catalyst system. A closer examination of the product distribution uncovered a non-monotonic trend in selectivity with respect to carbon chain length (Fig. 3E). The C_5_ olefin exhibited high aldehyde selectivity (97%), with only 3% isomeric olefins selectivity. As the carbon chain lengthened to C_7_ and C_8_, aldehydes selectivity dropped to 85%, alongside a 15% rise in internal olefins selectivity. Interestingly, further extension to C_10_ saw a recovery in aldehyde selectivity, reaching a level similar to that of C_5_. With further chain elongation, aldehyde selectivity dropped again and a concurrent increase in internal olefins selectivity, collectively giving rise to a distinct sinusoidal pattern. This trend was not apparent in final conversion, as all olefins achieved high conversion under the given conditions, minimizing observable activity differences. Therefore, we evaluated the activity of each olefin at low conversion. As shown in Fig. 3F, a parallel oscillatory trend was also observed. This sinusoidal variation in both olefin activity and aldehyde selectivity with increasing carbon chain length was not observed in other hydroformylation catalytic systems beyond Rh@MFI [13]. Given the potential impact of external mass-transfer limitations on hydroformylation activity and selectivity, a systematic investigation was therefore conducted by varying the stirring speed. As shown by the results (Table S11), external mass-transfer effects become negligible at speeds >600 r/min. Notably, all catalytic tests in this work were performed at 800 r/min, thereby minimizing the influence of external mass-transfer. Consequently, the observed chain-length oscillation phenomenon is not attributable to external mass-transfer effects. Such a unique phenomenon is therefore attributed to the confinement effect of the zeolite microenvironment. Notably, a similar chain-length-dependent oscillatory behavior has been reported for the diffusion of alkanes in zeolites, a process also known as resonant diffusion [32–34]. This similarity suggests a potential link between the observed catalytic behavior and the diffusion properties of alkenes within zeolites. Therefore, a thorough mechanistic investigation of the reaction matrix is desired to better understand the hydroformylation of long-chain *α*-olefins.

### Mechanistic study

In our prior study of the Rh-MEL zeolite catalyst, *in situ* infrared and X-ray absorption spectroscopies unequivocally revealed that the true active species under authentic hydroformylation conditions are encaged [RhH(CO)_2_] and [RhH(CO)] intermediates derived from single [Rh(CO)_2_] sites. This experimentally validated picture provided a robust foundation for DFT modeling, allowing us to map the complete Gibbs free energy profile for propylene hydroformylation and establish the first molecular-level structure–activity relationship for a zeolite-confined rhodium catalyst [8]. Although these calculations illuminated the intricate solid–liquid–gas interfacial mechanism within MEL micropores, they remained largely theoretical and awaited direct experimental corroboration. To rigorously validate the computed energy landscape, we conducted an integrated experimental strategy combining diffusion-reaction experiments, hydrogen (H)/deuterium (D) kinetic isotope effect measurements to interrogate rate-determining steps, *in situ* FT-IR monitoring of transient intermediates and site evolution, and selective passivation of external-surface rhodium using bulky thiol poisons (e.g. 2,4-dimethylbenzenethiol). We are confident that this synergistic approach will firmly ground theoretical predictions in observable catalytic behavior and elevate our understanding of confinement effects to a fully validated, molecularly precise level.

**Figure 4.**
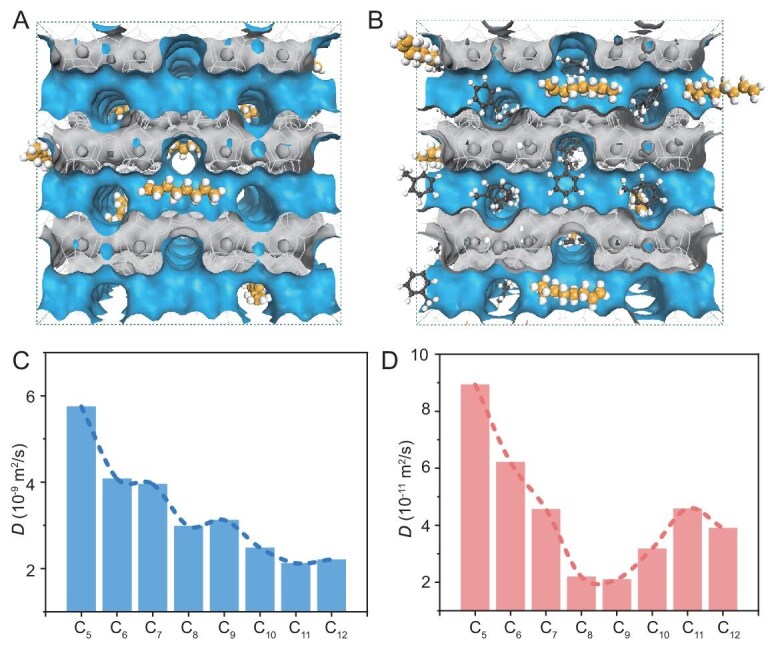
Diffusion of different olefin molecules within MEL channels. (A) Schematic model of a C_8_ olefin molecule inside MEL zeolite. (B) Schematic model of a C_8_ olefin and toluene molecules inside MEL zeolite. (C) Diffusion coefficient for various olefins inside MEL zeolite. (D) Diffusion coefficient of olefins co-adsorbed with toluene in MEL zeolite.

To our knowledge, no previous Rh-zeolite study has systematically investigated how *α*-olefin chain length modulates intrapore diffusion dynamics and, consequently, governs hydroformylation selectivity and activity—a critical yet overlooked dimension of confined catalysis. To bridge this knowledge gap, we employed molecular dynamic (MD) simulations to study the diffusion behavior of olefins with different chain lengths within the MEL channels. We investigated the diffusion behavior of individual olefins in MEL zeolite and found that the diffusion coefficients are on the order of 10^−9^ m^2^/s (Fig. 4A and C, Figs S15 and S16). As the molecular chain length increases, the diffusion coefficients generally decrease. Since diffusion is strongly solvent-dependent and our catalytic system contains abundant toluene, a solvent which itself can diffuse into the MEL zeolite channels, we reasoned that toluene would likely have a significant impact on olefin diffusion. Therefore, toluene molecules were included in the molecular dynamics simulations to better approximate the actual reaction environment. Notably, upon the introduction of toluene molecules, the diffusion coefficients exhibit a clear sinusoidal oscillation with increasing chain length, and ‘resonant diffusion’ becomes more evident (Fig. 4B and D). The overall diffusion coefficients decrease to ∼10^−11^ m^2^/s, indicating that toluene significantly restricts olefin diffusion while potentially enhancing the interaction between reactants and active sites in the zeolite. The more pronounced resonant diffusion observed when toluene molecules occupy the pores may be due to blocked channel intersections, leading to localized retention when the chain length of olefins matches the MEL pore dimensions (Figs S17 and S18). This resonant diffusion likely prolongs the retention time of olefins within the zeolite, thereby influencing reaction activity and product distribution, and giving rise to the sinusoidal pattern between the diffusion coefficient and chain length relationship [35,36]. It should also be noted that a slight discrepancy in chain length was observed between theoretical simulations and experimental data, which may arise from the gap between the idealized assumptions of the MD simulations and the inherent complexity of the real catalytic system. First of all, the actual reaction is dynamically complex. Reactants, aldehyde products, solvent, and syngas co-exist in this environment, where the generated aldehydes can remain in the pores and dynamically alter the effective pore size and diffusion pathways [31]. Furthermore, accurate diffusion modeling under working conditions presents significant challenges particularly in quantifying the distribution and precise local structure of active sites [37]. Despite deviations in peak positions, both MD simulations and experimental results demonstrate a highly consistent oscillatory trend with carbon chain length. Therefore, we conclude that olefin diffusion within the MEL zeolite is the primary factor governing the chain-length-dependent phenomenon.

**Figure 5.**
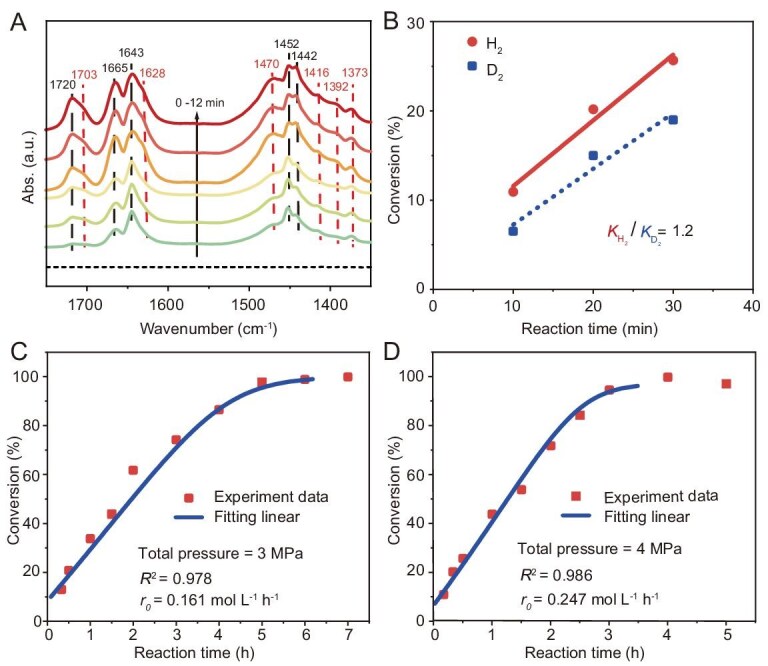
*In situ* IR characterization and kinetic study of propene hydroformylation over the Rh@MEL catalyst. (A) Time-dependent FT-IR spectra track the evolution of intermediate species under hydroformylation conditions (H_2_/CO/propene/Ar = 47:47:2:4, v/v/v/v; 4 MPa, 80°C). (B) H/D kinetic isotope effect (KIE, rateH_2_/rateD_2_) measurements for Rh@MEL-DMBT. (C and D) Experimental data points and fitted kinetic curves are shown as symbols and solid lines, respectively. Reaction conditions: 20 mg of catalyst, 4 mL of toluene, 80°C, CO/H_2_ = 1:1.

Considering the potential influence of diffusion kinetics on the reaction performance with long-chain olefins, propene was selected as the substrate to minimize diffusion limitations and thus allow for a clearer investigation of the microscopic reaction mechanism [38]. First, we sought direct spectroscopic evidence for the intermediates on the catalytic cycles for propene hydroformylation catalyzed by Rh@MEL-DMBT (Fig. S19). *In situ* IR spectroscopy can offer real-time vibrational information on organic intermediates formed during the reaction, thereby providing critical evidence into the reaction pathway. Therefore, we employed *in situ* IR spectroscopy to identify and monitor the formation and evolution of intermediates. Time-resolved IR spectra revealed that the initial CO vibrational bands at 2084 and 2007 cm^−1^ underwent a red shift to 2071 and 2003 cm^−1^, respectively. These shifted bands are assigned to the symmetric and asymmetric CO stretching vibrations of the [HRh(CO)_2_] species [39,40]. In addition, the concomitant emergence of a new band at 2036 cm^−1^ is ascribed to the active [HRh(CO)] hydride carbonyl species, formed via the dissociation of one CO molecule from the [HRh(CO)_2_] intermediate, which is consistent with previous *in situ* IR studies (Fig. S20) [8]. Concurrently, the growth of this band was accompanied by the appearance of four new absorption bands in the 1700–1750, 1628 and 1470 cm^−1^ region (Fig. 5A). This indicates that the active [HRh(CO)] species rapidly react with propene to form reactive intermediates or products. According to the literature, the band at 1720 cm^−1^ is assigned to the C=O stretching vibration of the product butyraldehyde adsorbed on Rh sites [17], while the shoulder at 1703 cm^−1^ corresponds to the C=O stretching vibration of a rhodium acyl intermediate [Rh(acyl) (CO)] [27,41–43]. Additional bands at ∼1445 and ∼1640 cm^−1^ are ascribed to gas-phase or zeolite-adsorbed propene [44]. The band at 1628 cm^−1^ is associated with the C=C stretching vibration of propylene adsorbed on the [HRh(CO)] site, indicating the formation of the [HRh(CO) (C_3_H_6_)] intermediate [17,39]. Furthermore, the band at 1470 cm^−1^, along with weaker features at 1416, 1392, and 1373 cm^−1^ is associated with −CH_2_ and −CH_3_ bending vibrations of Rh-alkyl or Rh-acyl species [27,44]. Collectively, these *in situ* IR results provide direct spectroscopic evidence for the formation of [HRh(CO) (C_3_H_6_)] and Rh-acyl intermediates, thereby corroborating the elementary steps of the hydroformylation mechanism on Rh@MEL-DMBT.

With the intermediates spectroscopically confirmed, we then addressed the significant question of the rate-determining step, which is of fundamental importance as it typically governs both the overall reaction activity and selectivity. The increasing intensity of the infrared peaks associated with the [HRh(CO) (C_3_H_6_)] or [Rh(acyl) (CO)] intermediate suggests that a subsequent step—either an alkene insertion step or the oxidative addition/reductive elimination involving the Rh-acyl species step—could result in a slow reaction. Moreover, numerous studies have demonstrated that these steps (e.g. olefin insertion step or CO insertion step) could all potentially serve as rate-determining steps, depending on the reaction conditions and catalyst composition [45–47]. To discriminate among these possibilities, we performed H/D kinetic isotope effect experiments. Steps involving H–H bond cleavage or hydride transfer typically exhibit significant primary kinetic isotope effect (KIE) (*k*_H2_/*k*_D2_ > 2) [48]. As shown in Fig. 5B, substituting D_2_ for H_2_ led to only a modest rate decrease, yielding a KIE value of 1.2. The relatively low KIE values suggest that hydrogen-involving elementary steps are unlikely to be the rate-determining step in the catalytic cycle [49].

Having ruled out the hydrogen-involving elementary steps, we therefore turned to evaluate the potential of CO insertion as the rate-determining step by performing a kinetic model study. Applying the equilibrium hypothesis, we derived the rate equation and the conversion-time equation considering CO insertion as the rate-determining step (Note S1) [50]. Under our experimental conditions, rate equation simplifies to a power-law dependence of the initial rate (*r_0_*) on total pressure (*p*), *r*_0_ ∝ *p*^1.5^. For comparison, we also modeled the scenario where the alkene insertion step was treated as the rate-determining step. Based on the same derivation process, the relationship between *r_0_* and *p* was obtained as *r_0_* ∝ *p*^0.5^. With these distinct theoretical predictions, the pressure dependence of the initial rate was experimentally determined to validate the hypothesis and unambiguously assign the rate-determining step. We measured propene conversion as a function of time at different pressures and fitted the data to the conversion-time equation. The model excellently describes the experimental data (*R*^2^ > 0.97, Fig. 5C and D), with calculated initial rates of 0.161 and 0.247 mol L^−1^ h^−1^ at 3 and 4 MPa, respectively (Table S12). Further logarithmic analysis of *r_0_* and *p* revealed that the initial rate follows a power-law relationship with total pressure, *r_0_* ∝ *p*^1.49^. The relationship between *r_0_* and *p* obtained from the experiment aligns most closely with the exponent of 1.5 predicted for the CO insertion step as the rate-determining step. This indicates that the CO insertion step is most likely to be the rate-determining step. In summary, the synergistic combination of kinetic isotope effects and detailed kinetic modeling provides compelling experimental evidences for the carbonyl insertion step as the rate-determining step in the Rh@MEL-DMBT catalyzed hydroformylation of propene.

## CONCLUSIONS

By selectively passivating external rhodium sites with a bulky thiol, we have successfully isolated the intrinsic catalytic behavior of single Rh centers confined within MEL zeolite micropores, thereby eliminating long-standing ambiguities arising from coexisting unrestricted surface species. The resulting Rh@MEL-DMBT catalyst exhibits extraordinary shape-selective hydroformylation of higher *α*-olefins (C_5_–C_12_), delivering linear-to-branched aldehyde ratios up to 601, >95% aldehyde chemoselectivity, and virtually complete suppression of isomerization—performance unmatched by conventional homogeneous or heterogeneous systems. A striking sinusoidal dependence of activity and aldehyde selectivity on substrate chain length emerges, which we trace directly to resonant diffusion of *α*-olefins in solvent-filled MEL channels through molecular dynamics simulations. Combined *in situ* FT-IR spectroscopy, deuterium KIE, and pressure-dependent kinetic modeling unambiguously identify CO insertion into the Rh-alkyl bond as the rate-determining step and confirm the active intermediates as encaged [HRh(CO)_2_] and [HRh(CO)] species. These findings establish the first fully experimentally validated molecular-level mechanism for zeolite-confined single-site rhodium hydroformylation and reveal a hitherto unrecognized interplay between intrapore diffusion dynamics and catalytic outcomes. Beyond resolving a decades-old challenge in regioselective olefin functionalization, this work illuminates a general design principle—that spatial confinement can orchestrate both thermodynamic and kinetic selectivity through substrate-specific resonant diffusion—opening new avenues for precision catalysis in confined environments.

## METHODS

### Preparation of Rh@MEL materials

The preparation of the Rh@MEL catalyst in this work closely follows our previously reported procedure for encapsulating Rh species within an MEL zeolite [8]. We herein focus on evaluating a higher Rh loading (0.32 wt%) for the hydroformylation of long-chain *α*-olefins. In the representative synthesis, the Rh@MEL material was synthesized through the following procedure. Initially, 0.03 g of KOH (0.5 mmol) was dissolved in 6.2 g of tetrabutylammonium hydroxide (TBAOH, 6.0 mmol), followed by the addition of 4.12 g of tetraethyl orthosilicate (TEOS, 19.8 mmol) to obtain a homogeneous hydrolyzed solution. A metal precursor solution was prepared by dissolving 9.0 mg of RhCl_3_·xH_2_O (0.034 mmol) and 100 μL of ethylenediamine in 100 μL of deionized water. Under vigorous stirring, the metal precursor solution was added dropwise to the hydrolyzed solution. The resulting mixture was stirred at 30°C for 10 hours (500 r/min) to complete the hydrolysis process. The pale yellow mixture was then transferred into a 50-mL Teflon-lined autoclave and heated statically at 130°C for 24 hours. After hydrothermal treatment, the solid product was collected by centrifugation and washed repeatedly with deionized water and ethanol. The obtained solid was dried at 80°C in air for 12 hours. Finally, the sample was calcined in air at 560°C for 5 hours, followed by reduction under a H_2_/Ar mixture at 600°C for 2 hours.

### Catalytic evaluations

For substrates that are liquid at atmospheric pressure, the detailed experimental procedure is as follows: the Rh@MEL catalyst (20 mg, 0.32 wt% Rh), a linear *α*-olefin (1 mmol), 2 mg of DMBT, and toluene (4 mL) were added to a 12-mL threaded vial equipped with a magnetic stir bar. The vial was sealed with a threaded mid-hole cap and connected to the external atmosphere via a needle. One or more such vials containing the reaction mixture were then placed into a 250-mL high-pressure autoclave. The autoclave was purged three times with CO and subsequently charged with CO (2 MPa) and H_2_ (2 MPa). After the oil bath temperature had stabilized at 80°C, the sealed autoclave was immersed into it. The reaction was then conducted at this temperature with stirring at 800 r/min. for 1 hour. After the reaction, the autoclave was cooled to room temperature, and the pressure was released to atmospheric pressure. Subsequently, 100 μL of 1-nonane was added as an internal standard to the reaction mixture, followed by gas chromatographic analysis (Agilent 8890, equipped with an HP-5 column).

## Supplementary Material

nwag110_Supplemental_File
