# Supplementary material for: Unmasking true confinement effects: ultrahigh linear selectivity and chain-length oscillatory behavior in zeolite-encapsulated rhodium hydroformylation
Source: Natl Sci Rev. 2026 Feb 23;13(9):nwag110. doi: 10.1093/nsr/nwag110 (PMC13182251; doi:10.1093/nsr/nwag110)
Supplement: nwag110_Supplemental_File [file nwag110_supplemental_file.pdf]

Supporting Information for

Unmasking true confinement effects: ultrahigh linear selectivity and chain-length oscillatory behavior in zeolite-encapsulated rhodium hydroformylation

Tao Yan[1,2,3,†], Xiangjie Zhang[2,†], Gengzhe Song[2,†], Ziyu Zhou[2], Huaming Hou[2], Qingqing Xie[1,2,3], Huizi He[1,2,3], Xin Tong[1,2,3], Hongying Chang[3], Zhiqiang Liu[4,*], Anmin Zheng[4], Zhi Cao[1,2,3,*], Peng He[1,2,3,*]

[1]State Key Laboratory of Coal Conversion, Institute of Coal Chemistry, Chinese Academy of Sciences, Taiyuan 030001, China

[2]National Energy Center for Coal to Clean Fuels, Synfuels China Technonology Co., Ltd., Beijing 101407, China

[3]University of Chinese Academy of Sciences, Beijing 100190, China

[4]Interdisciplinary Institute of NMR and Molecular Sciences, Key Laboratory of Hubei Province for Coal Conversion and New Carbon Materials, School of Chemistry and Chemical Engineering, Wuhan University of Science and Technology, Wuhan 430081, China

[†]These authors contributed equally to this work

*Corresponding author. Email: zqliu_wh@wust.edu.cn (Z.L.); caozhi@sxicc.ac.cn (Z.C.); hepeng@sxicc.ac.cn (P.H.)

Chemicals and Materials

Tetraethyl orthosilicate (TEOS, 99.9%, Sigma-Aldrich), 1-Pentene (≥98.5%, Sigma-Aldrich), 1-Hexene (97%, Sigma-Aldrich), 1-Heptene (97%, Sigma-Aldrich), 1-Octene (≥99%, Alfa Aesar), 1-Nonene (96%, Alfa Aesar), 1-Decene (97%, Alfa Aesar), 1-Undecene (97%, Sigma-Aldrich), 1-Dodecene (96%, Alfa Aesar), 1-Tridecene (97%, Alfa Aesar), 2-hexene (96%, Alfa Aesar), 3-hexene (97%, Alfa Aesar), 2-octene (96%, Alfa Aesar), Toluene (analytical grade, Sinopharm), $RhCl_3 \cdot xH_2O$ (≥98%, Innochem), Ethylenediamine (≥99.5%, Sigma-Aldrich), Tetrabutylammonium hydroxide (25%, Innochem), KOH (80%, Sigma-Aldrich), *n*-Nonane (99.8%, Sigma-Aldrich), 2,4-Dimethylbenzenethiol (96%, Innochem), 1-Propanethiol (98%, Alfa), deionized (DI) water (>18.2 MΩ cm^{-1}). All chemicals were used without further purification.

Preparation of Rh@MEL materials

The preparation of the Rh@MEL catalyst in this work closely follows our previously reported procedure for encapsulating Rh species within an MEL zeolite[1]. We herein focus on evaluating a higher Rh loading (0.32 wt%) for the hydroformylation of long-chain α-olefins. In the representative synthesis of the Rh@MEL material was synthesized through the following procedure. Initially, 0.03 g of KOH (0.5 mmol) was dissolved in 6.2 g of tetrabutylammonium hydroxide (TBAOH, 6.0 mmol), followed by the addition of 4.12 g of tetraethyl orthosilicate (TEOS, 19.8 mmol) to obtain a homogeneous hydrolyzed solution. A metal precursor solution was prepared by dissolving 9.0 mg of $RhCl_3 \cdot x$H2O (0.034 mmol) and 100 μL of ethylenediamine in 100 μL of deionized water. Under vigorous stirring, the metal precursor solution was added dropwise to the hydrolyzed solution. The resulting mixture was stirred at 30 ℃ for 10 hours (500 r.p.m.) to complete the hydrolysis process. The pale yellow mixture was then transferred into a 50 mL Teflon-lined autoclave and heated statically at 130 ℃ for 24 hours. After hydrothermal treatment, the solid product was collected by centrifugation and washed repeatedly with deionized water and ethanol. The obtained solid was dried at 80 ℃ in air for 12 hours. Finally, the sample was calcined in air at 560 ℃ for 5 hours, followed by reduction under a H_2/Ar mixture at 600 ℃ for 2 hours. Using this method, we statistically analyzed six synthesized batches of the catalyst. The obtained catalyst mass ranged from 0.94 to 1.03 g, corresponding to a synthesis yield of 78.3% to 85.8% based on the moles of SiO_2, confirming good reproducibility. Furthermore, the scalability was verified by scaling up the synthesis by a factor of 10, which afforded the catalyst in a yield of 79.5%. This result indicates no significant issues in the preliminary scale-up

preparation.

Catalyst Characterization

The Rh content in synthesized materials was determined by inductively coupled plasma optical emission spectrometry (ICP-OES) using an Optima 2100 DV spectrometer (PerkinElmer, USA).

Powder X-ray diffraction (PXRD) measurements were performed at ambient temperature on a Bruker D8 ADVANCE diffractometer (Bruker AXS, Germany) equipped with a Cu Kα radiation source (λ = 1.5406 Å). The instrument was operated at 40 kV and 40 mA, and the diffraction patterns were collected in step-scan mode across the 2θ range of 5-90 ° with a step size of 0.02 °. The diffractometer was calibrated using LaB6 standard powder (NIST SRM 660c) prior to measurements to ensure accurate angular positioning.

The atomic-scale structural characterization was performed using aberration-corrected high-angle annular dark-field scanning transmission electron microscopy (HAADF-STEM). The measurements were conducted using two advanced microscopes: a FEI Titan Cubed Themis G2 and a JEOL JEM-ARM300F, both operated at an acceleration voltage of 300 kV. High-resolution images were acquired with two different pixel resolutions: 2,048 × 2,048 and 1,024 × 1,024, employing a variable dwell time ranging from 3 to 12 μs per pixel to optimize signal-to-noise ratio. The beam current was carefully maintained below 10 pA to minimize potential beam damage to the samples.

Complementary elemental mapping was performed using energy-dispersive X-ray spectroscopy (EDS) in a FEI Talos transmission electron microscope operated at 200 kV, enabling simultaneous acquisition of structural and compositional information at the nanoscale.

Fourier-transform infrared spectroscopy (FT-IR) analysis was conducted using a Bruker VERTEX 70v spectrometer equipped with a liquid nitrogen-cooled mercury cadmium telluride (MCT) detector. All spectra were acquired in transmission mode with a spectral resolution of 4 cm^{-1}, accumulating 64 scans to enhance signal quality, across the wavenumber range of 1,000-4,000 cm^{-1}. For sample preparation, approximately 20 mg of the material was pressed into a self-supported wafer (13 mm diameter) and mounted in a high-temperature infrared cell featuring BaF_2 windows, which was integrated with a vacuum-adsorption system for precise pressure regulation. Prior to adsorption measurements, the catalyst samples were first reduced under flowing H_2 at 500 ℃ for 1 h, purged with He for 30 min, and then cooled to 25 ℃ under He atmosphere. CO adsorption studies were subsequently conducted by gradually increasing the CO pressure in the IR cell while continuously

monitoring the infrared spectrum until spectral stabilization was achieved. The thiol poisoning process was monitored by collecting infrared spectra during exposure of the Rh@MEL samples to DMBT or propanethiol vapor (5 mbar), using CO as the carrier gas. For *in situ* FT-IR measurements under reaction conditions, the samples were loaded into a custom-designed infrared cell with a 200 μm optical path length. The samples were exposed to a reactant gas mixture (H_2/CO/propene/Ar = 47:47:2:4, v/v/v/v) at 80 ℃ and 4 MPa total pressure. To distinguish the Rh carbonyl signals from gaseous CO interference, spectral subtraction was performed using a previously established data processing methodology, ensuring accurate identification of surface-adsorbed CO species.

The ^{29}Si solid-state MAS NMR experiments were performed at 9.4 T on a Bruker Avance III 400 spectrometer at resonance frequencies of 79.33 MHz. Single-pulse ^{29}Si MAS NMR spectra with high power proton decoupling were recorded on a 7 mm probe, using a π/2 pulse of 5.2 μs, a recycle delay of 60 s and 128 scans. The magic angle spinning rate for ^{29}Si MAS NMR was set to 6 kHz. The ^{29}Si MAS NMR spectra were referenced to kaolinite (-91.5 ppm).

X-ray absorption spectroscopy (XAS) measurements at the Rh K-edge were performed at the BL14W1 and BL13SSW beamlines of the Shanghai Synchrotron Radiation Facility (SSRF). A Si (311) double-crystal monochromator was employed for energy selection, and data acquisition was conducted in fluorescence yield mode using a four-element germanium (Ge) detector. Energy calibration was performed using a rhodium foil reference, with the absorption edge energy defined as the first inflection point of the derivative spectrum. Data processing and analysis were carried out using the ATHENA and ARTEMIS software packages. Theoretical models for R-space fitting were generated using FEFF calculations based on the crystal structures of Rh metal and Rh_2O_3. For XAS measurements, the samples were pressed into uniform flakes (5 mm thickness) and loaded into custom-designed sample cell.

The textural properties of the Rh@zeolite catalysts were characterized by N_2 physisorption measurements using a Micromeritics ASAP 2020 analyzer. The specific surface area was calculated using the Brunauer-Emmett-Teller (BET) method, while the pore size distribution was determined through the Barrett-Joyner-Halenda (BJH) analysis of the desorption branch. The total pore volume was evaluated by the t-plot method at a relative pressure (P/P_o) of 0.99.

Morphological characterization was performed using scanning electron microscopy (SEM, FEI QUANTA 400F) operated at an acceleration voltage of 10 kV, enabling the examination of zeolite

particle morphology and size distribution.

Surface chemical analysis was conducted using X-ray photoelectron spectroscopy (XPS) on a Physical Electronics Quantum-2000 Scanning ESCA Microprobe system equipped with monochromatic Al Kα radiation (hν = 1486.6 eV). Prior to analysis, samples were pretreated by reduction in H_2 atmosphere at 600 ℃ for 1 hour and transferred to the analysis chamber under oxygen-free conditions using a glovebox. The analysis chamber was maintained at an ultra-high vacuum ($<10^{-10}$ Pa) during measurements. Energy calibration was performed using the C 1s peak of adventitious carbon at 284.8 eV as an internal reference.

Catalytic evaluations

For substrates that are liquid at atmospheric pressure, the detailed experimental procedure is as follows: The Rh@MEL catalyst (20 mg, 0.32 wt% Rh), a linear α-olefin (1 mmol), 2 mg of DMBT, and toluene (4 mL) was added to a 12 mL threaded vial equipped with a magnetic stir bar. The vial was sealed with a threaded mid-hole cap and connected to the external atmosphere via a needle. One or more such vials containing the reaction mixture were then placed into a 250 mL high-pressure autoclave. The autoclave was purged three times with CO and subsequently charged with CO (2 MPa) and H_2 (2 MPa). After the oil bath temperature had stabilized at 80 ℃, the sealed autoclave was immersed into it. The reaction was then conducted at this temperature with stirring at 800 r.p.m. for 1 hour. After the reaction, the autoclave was cooled to room temperature, and the pressure was released to atmospheric pressure. Subsequently, 100 μL of 1-nonane was added as an internal standard to the reaction mixture, followed by gas chromatographic analysis (Agilent 8890, equipped with an HP-5 column). Considering the potential issue of temperature inhomogeneity when using multiple vials inside a single autoclave, triplicate experiments should be conducted at various positions. Data points that were significantly higher or lower were excluded as outliers. Positions yielding consistent results were identified and subsequently fixed for all following tests to ensure experimental reproducibility. The TOF was defined as the moles of aldehyde produced per hour to the total moles of Rh present in the catalyst, calculated according to the following equation:

TOF = (Moles of aldehydes)/(Total moles of Rh in the catalyst × reaction time)

For the TOF measurements with different olefin substrates, the reaction time was adjusted to maintain the conversion at approximately 10%. Each measurement was performed in triplicate under identical conditions to ensure data reproducibility.

Scale-up experiments were conducted according to the following detailed procedure. The Rh@MEL catalyst (200 mg, 0.32 wt% Rh), a linear α-olefin (10 mmol), 20 mg of DMBT, and toluene (40 mL) were added to a 250 mL high-pressure autoclave equipped with a magnetic stir bar. The autoclave was purged three times with CO and subsequently charged with CO (2 MPa) and H_2 (2 MPa). After the oil bath temperature had stabilized at 80 ℃, the sealed autoclave was immersed into it. The reaction was then conducted at this temperature with stirring at 800 r.p.m. for 1 hour. After the reaction, the autoclave was cooled to room temperature, and the pressure was released to atmospheric pressure. Subsequently, 1000 μL of 1-nonane was added as an internal standard to the reaction mixture, followed by gas chromatographic analysis (Agilent 8890, equipped with an HP-5 column).

The hydroformylation of propylene was carried out in a 25 mL high-pressure reactor equipped with electric heating. The reactor has an internal diameter of 28 mm and a height of 42 mm. The detailed experimental procedure is as follows: 20 mg of catalyst, 4 mL of toluene, 2 mg of DMBT and a magnetic stir bar were loaded into the reactor. The reaction gas mixture (H_2/CO/propylene/Ar, 47:47:2:4, v/v/v/v) was introduced to purge the system three times. The reaction was then conducted at 80 ℃ with stirring at 800 r.p.m. for 1 hours. After the reaction, the reactor was cooled to room temperature and carefully depressurized to atmospheric pressure. Subsequently, 100 μL of 1-nonane was added as an internal standard to the reaction mixture, followed by centrifugation at 10,000 r.p.m. to collect the liquid phase. The liquid phase was analyzed using an Agilent 8890 gas chromatograph equipped with an HP-5 capillary column and an FID detector. The tail gas collected after the reaction was analyzed using the same Agilent 8890 GC system with thermal conductivity and FID detectors. Gases (Ar, CO, and H_2) and hydrocarbons (propylene, propane) in the tail gas were separated using HP-5MS (30 m × 0.25 mm × 0.25 μm) and GasPro (30 m × 0.32 mm × 0.32 μm) capillary columns, respectively. The conversion of propylene was determined by analyzing the concentration changes of propylene in both gaseous and liquid mixtures, using Ar and n-nonane as internal standards.

The carbon balance calculation equation based on the 1-hexene is as follows:

$$\text{Carbon balance}(\%) = \frac{N_{unconverted\ 1-hexene} + N_{heptanal} + N_{internal\ alkenes} + N_{hydrogenation\ byproducts}}{N_{feed\ of\ 1-hexene}} \times 100\%$$

where $N_{unconverted\ 1\text{-}hexene}$ is the moles of unconverted 1-hexene remaining after the reaction, $N_{heptanal}$ is the total moles of all heptaldehyde isomers produced during the reaction, $N_{internal\ alkenes}$ is the total

moles of internal alkenes formed via the isomerization of 1-hexene during the reaction, $N_{hydrogenation\ byproducts}$ is the total moles of hexane and heptanol generated from the hydrogenation of hexene and heptanal during the reaction and $N_{feed\ of\ 1\text{-}hexene}$ is the initial moles of 1-hexene charged into the reactor.

The catalytic stability of Rh@MEL-DMBT was evaluated over consecutive reaction cycles. Following each run, the spent catalyst was separated by centrifugation, washed with toluene three times, and then followed by calcination in static air at 560 ℃ for 2 h and then reduction by H_2/Ar mixture at 500°C for 1 h.

The encapsulation efficiency of the Rh@MEL samples was evaluated through hydrogenation reactions using 1-decene or cyclooctene as the substrate[2]. In a typical procedure, the autoclave was charged with the substrate, purged four times with hydrogen, and then pressurized to 4 MPa. The reaction was conducted at 70°C with stirring at 800 r.p.m. for 20 minutes, after which the autoclave was cooled in an ice bath. The resulting mixture was separated by centrifugation and mixed with 100 μL of 1-nonane as an internal standard. Quantitative analysis was performed using an Agilent 8890 gas chromatography system equipped with an HP-5 capillary column. To ensure that the reaction rates were measured in the kinetic regime, the conversion of 1-decene or cyclooctene was maintained below 10% by adjusting the reaction time, thereby minimizing mass transfer effects.

Molecular dynamics (MD) simulation

Two simulation models were constructed. Model A (Fig. 4A) represented a system of pure alkene molecules (C_5–C_{12}) within the pores of an MEL zeolite (Fig. S15), and the atoms of zeolites were rigid. Model B (Fig. 4B) incorporated both the alkenes and toluene solvent under confinement (Figure S16), and all the atoms of zeolites were flexible. The initial framework structures of pure silicon MEL were taken from the International Zeolites Associations (IZA) database. A 2×2×3 supercell (the lattice parameters were 40.540 × 40.540 × 40.377 Å^3) was constructed, with loadings of 12 alkene molecules (Model A) and 12 alkene / 48 toluene molecules (Model B), respectively.

MD simulations were performed in the canonical ensemble (NVT), where the number of particles (N), volume (V), and temperature (T) were kept constant. The simulated temperature was 353 K and controlled by a Nosé-Hoover thermostat with a coupling time constant of 0.1 ps. The leapfrog Verlet algorithm was used to integrate the Newton's equations of motion with a time step of 1.0 fs. Each MD simulation was equilibrated over 1×10^5 steps, and then the following 2×10^7 steps were used to study the diffusion behaviors of adsorbate molecules. The COMPASS force field was used and the cutoff

radius was 12.5 Å.[3-5] The trajectories were recorded every 1000 steps, and 3 independent MD simulations were conducted for better statistics.

The mean square displacement (MSD, Fig. S19) of alkanes is defined by the following equation:

$$MSD(\tau) = \frac{1}{N_m}\sum_{i}^{N_m}\frac{1}{N_\tau}\sum_{t_0}^{N_\tau}[r_i(t_0+\tau) - r_i(t_0)] \qquad (1)$$

where N_m N_m is the number of gas molecules, N_τ is the number of time origins used to calculate the average, and r_i is the coordinate of the *i*-th molecule. The self-diffusion coefficient, D_s, may be determined by calculating the slope of the Einstein relation describing the variation in *MSD* as a function of time, as shown in equation (2):

$$MSD(\tau) = 6D_s\tau + b \qquad (2)$$

The D_s values reported herein were calculated as the average of 3 dependent MD trajectories. Simulations were performed with Forcite module in the Material Studio software (Accelrys Software Inc., San Diego, CA).

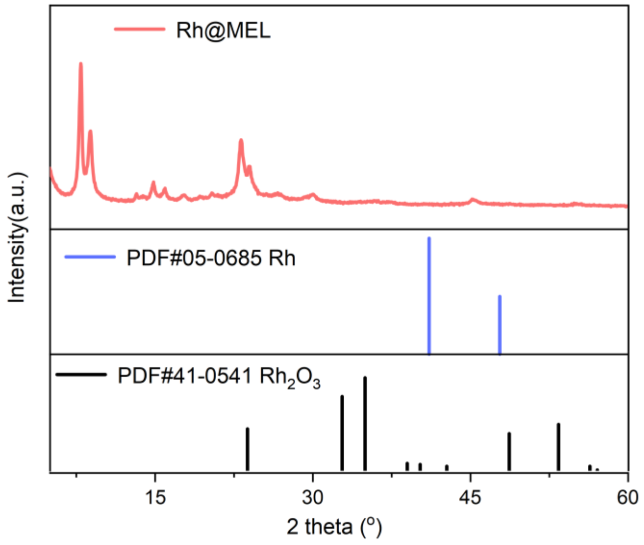

Figure S1. XRD patterns of Rh@MEL catalysts. The preserved characteristic diffraction peaks verify the maintenance of MEL crystalline topology after Rh encapsulation.

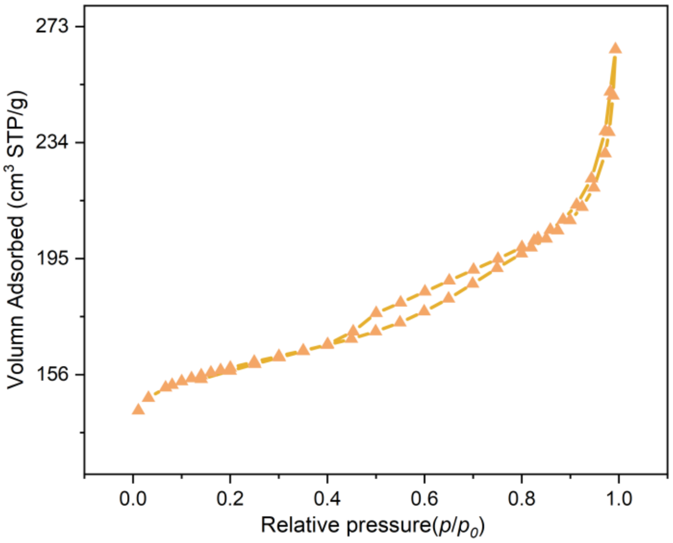

Figure S2. N_2 Adsorption-Desorption Isotherms of Rh@MEL catalysts.

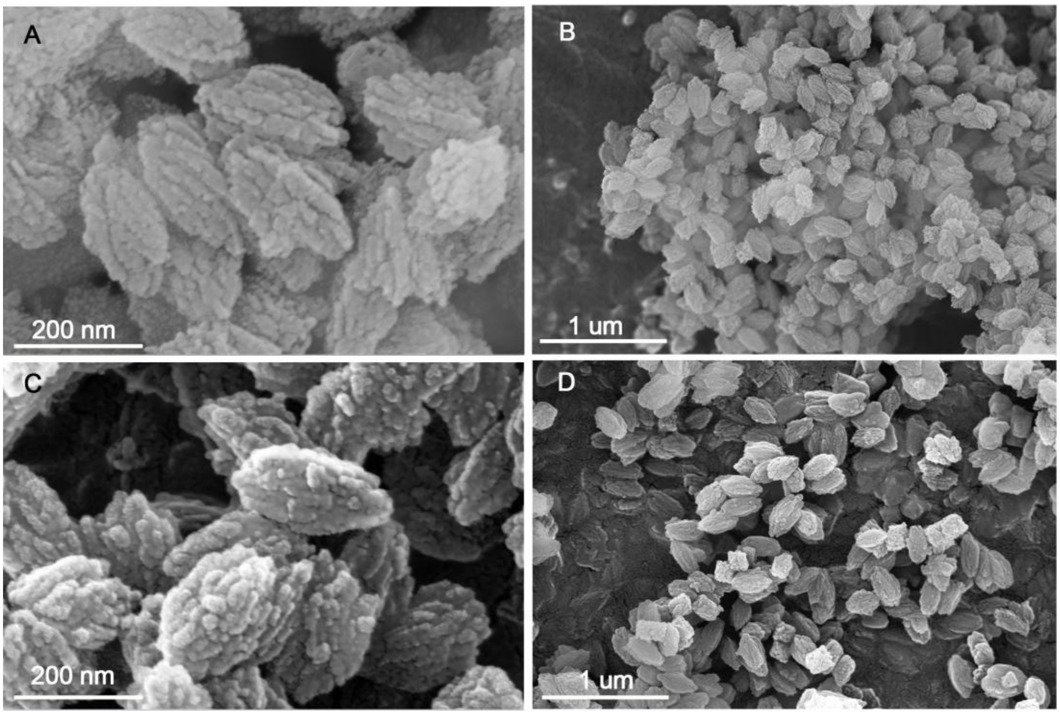

Figure S3. Representative SEM imaging of Rh@MEL.

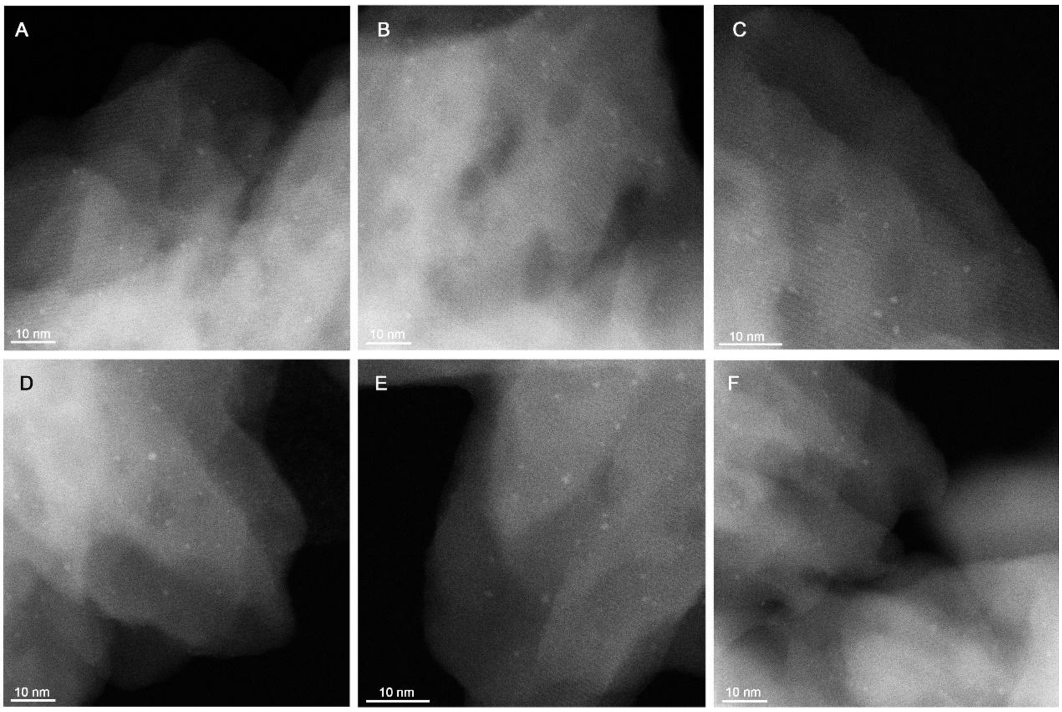

Figure S4. Representative aberration-corrected HAADF-STEM imaging of Rh@MEL. Uniform dispersion of Rh clusters within the zeolite.

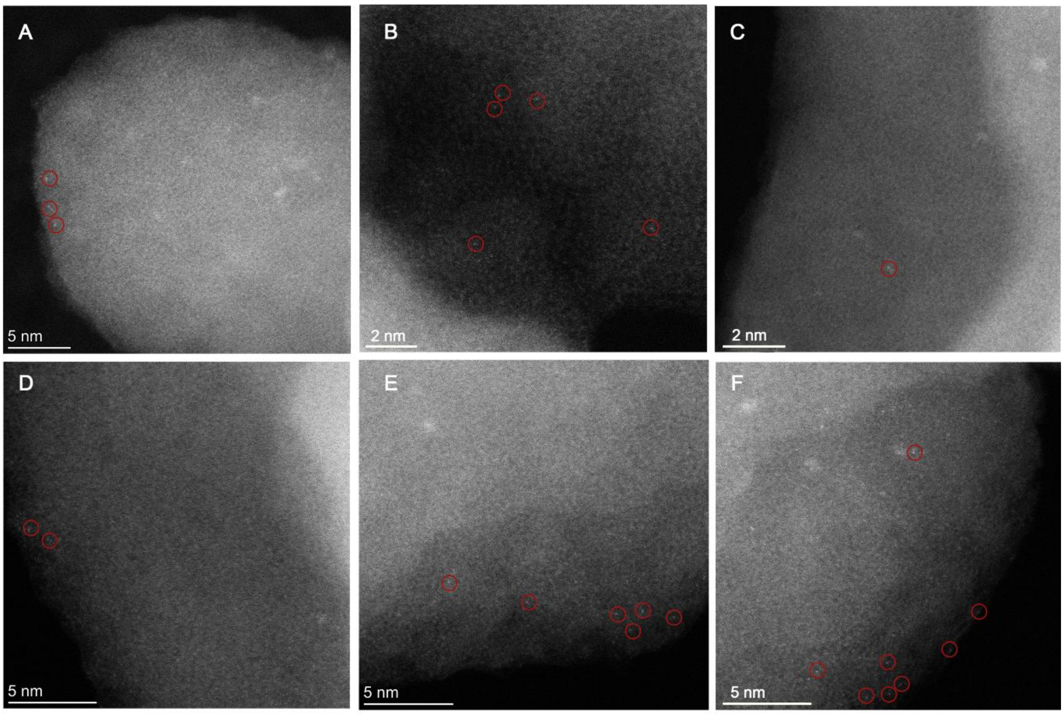

Figure S5. Representative aberration-corrected HAADF-STEM imaging of Rh@MEL. Atomic-resolution image identifying single-atom Rh species (highlighted in red circles).

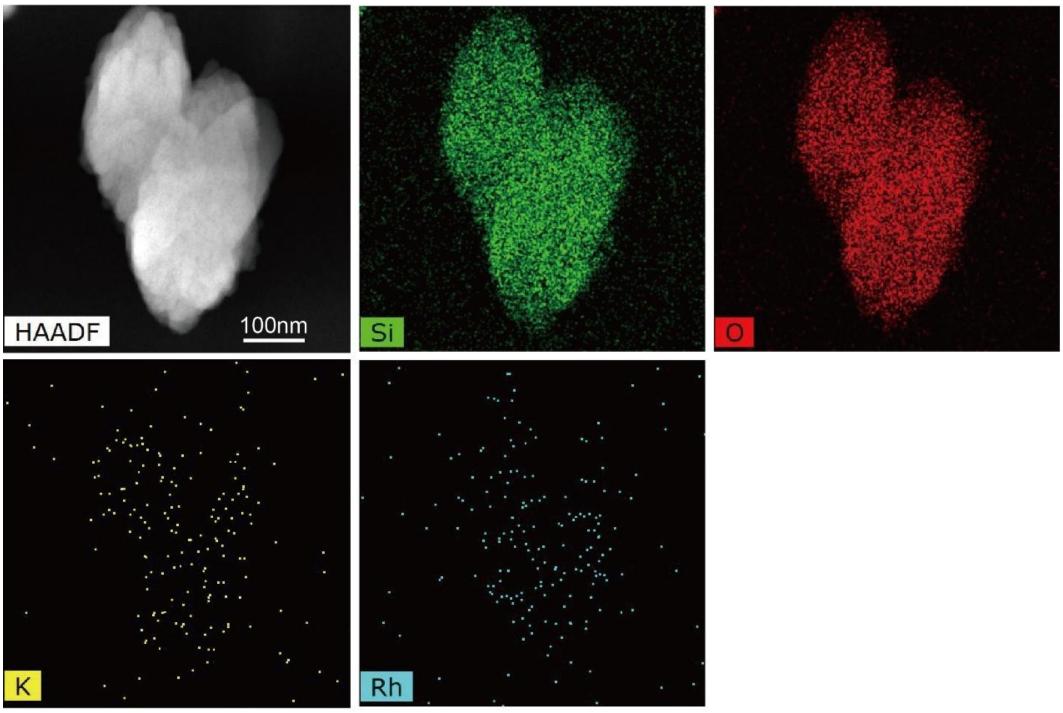

Figure S6. Representative HAADF-STEM images and corresponding EDX elemental maps of Rh@MEL. Elemental distributions of Si (green), O (red), K (yellow), and Rh (blue) confirm the homogeneous dispersion of Rh species throughout the MEL zeolite crystallites.

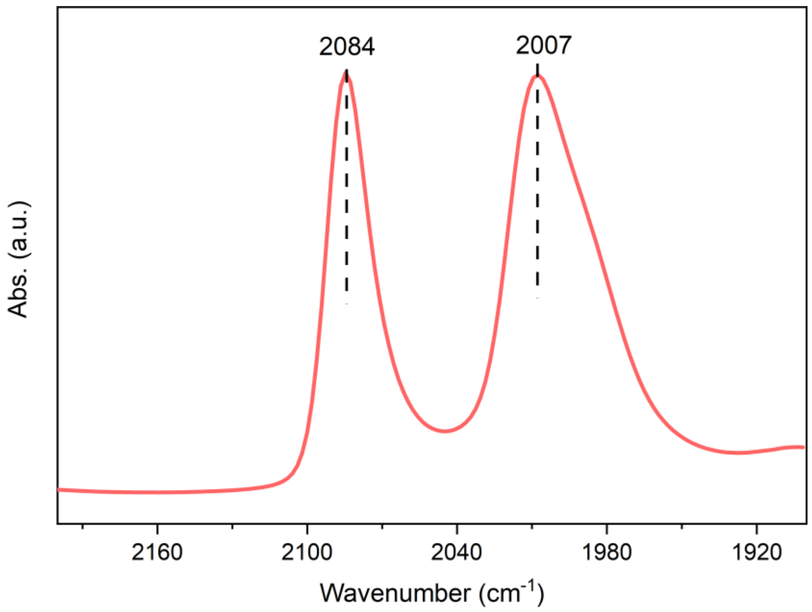

Figure S7. FT-IR spectra of CO adsorbed on the Rh@MEL. The IR absorption bands at 2084 and 2007 cm^{-1} can be attributed to the symmetric and asymmetric stretching vibrations, respectively, of geminal dicarbonyl $Rh(CO)_2$ species.[6,7]

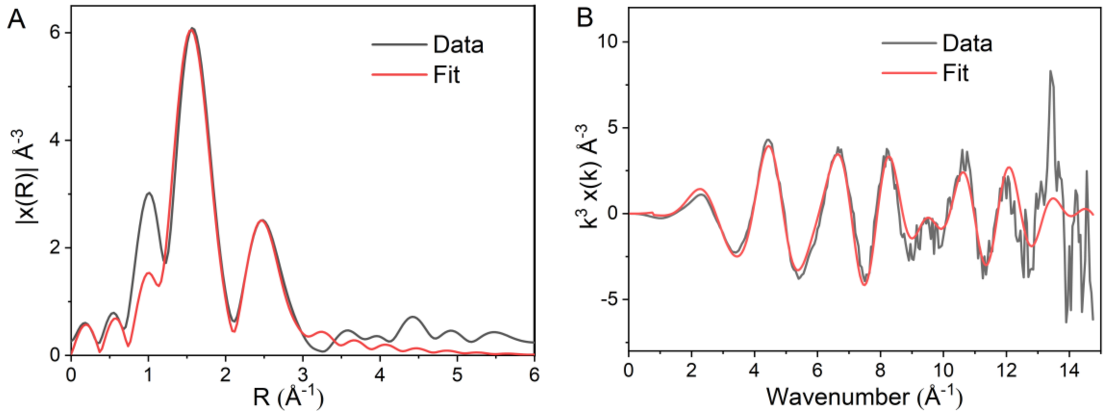

Figure S8. Results of the fitting of the EXAFS spectra of Rh@MEL catalysts. FT-EXAFS spectra with fitting results for Rh@MEL (A) and k^3-weighted EXAFS oscillations and theoretical fit for Rh@MEL (B).

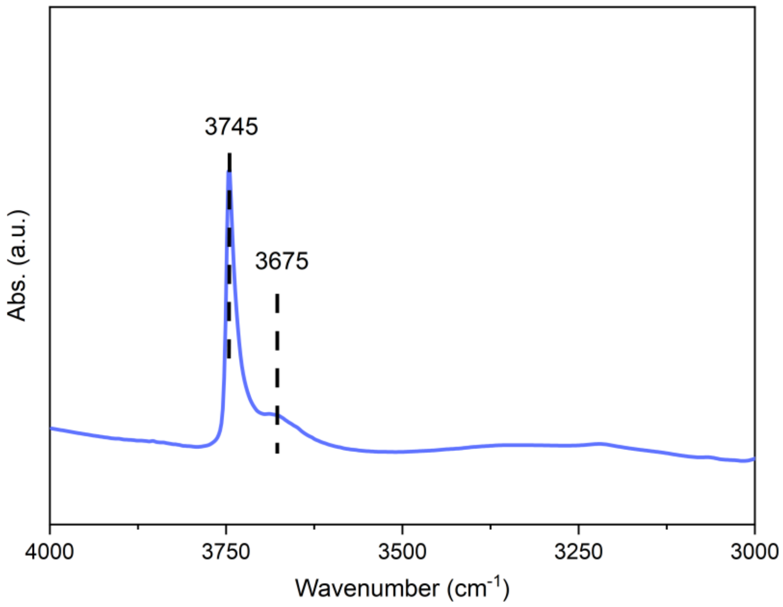

Figure S9. IR spectra of Rh@MEL in the 4000-3000 cm^{-1} range. The well-defined peaks observed at 3745 cm^{-1} and 3675 cm^{-1} in the IR spectra are unequivocally assigned to isolated silanols (≡Si-OH) and hydrogen-bonded silanols (≡Si-OH···O≡Si), respectively.[8,9]

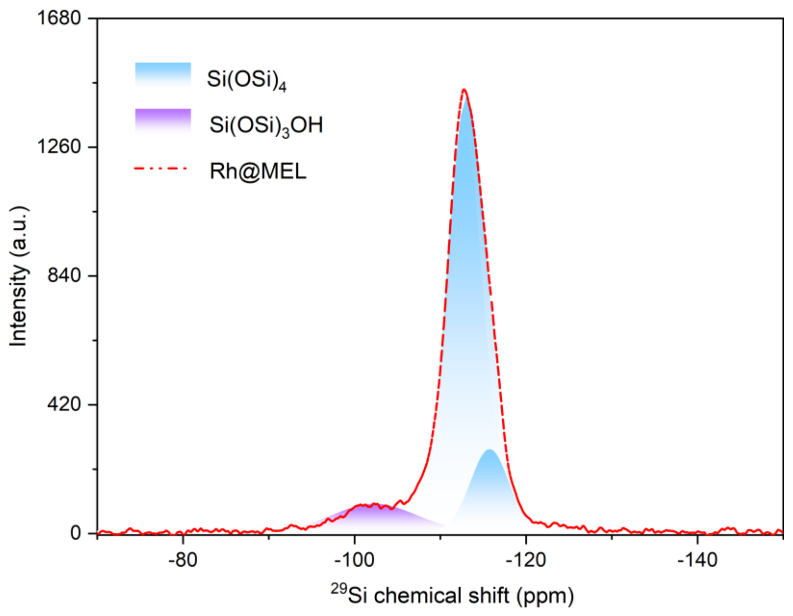

Figure S10. ^{29}Si solid-state NMR spectra of Rh@zeolites. In addition to the dominant Q^4 [$Si(OSi)_4$] environments, the ^{29}Si NMR spectra of Rh@MEL reveal the presence of Q^3 [$Si(OSi)_3OH$] sites, indicating partial framework defects.[9]

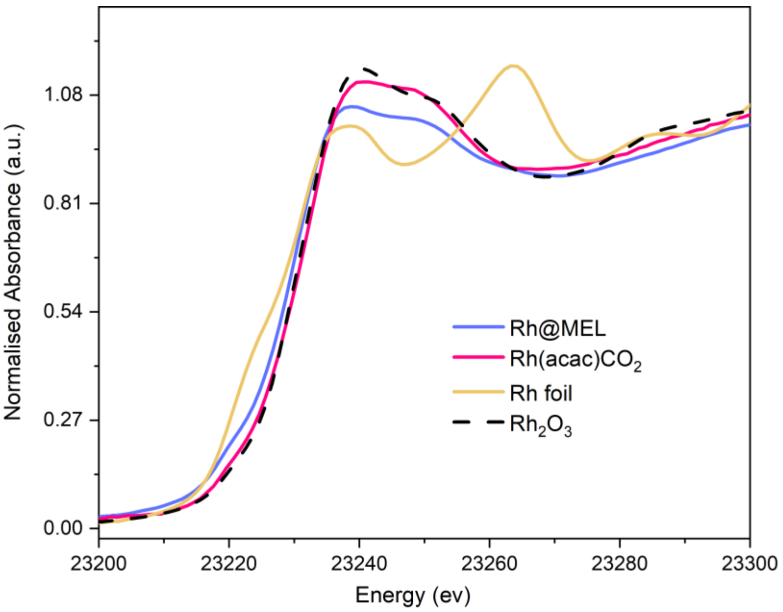

Figure S11. Normalized Rh K-edge XANES spectra

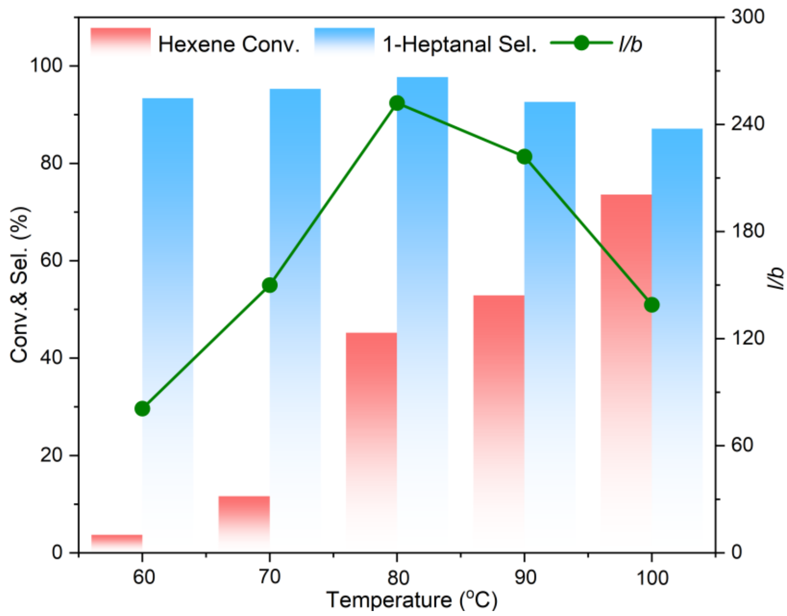

Figure S12. The optimization of the reaction temperature over the Rh@MEL-DMBT catalyzed hydroformylation. Reaction conditions: 20 mg of catalyst, 1 mmol hexene, 4.0 mL of toluene, 4 MPa of H_2/CO (1:1, v/v), 800 r.p.m., 1 h.

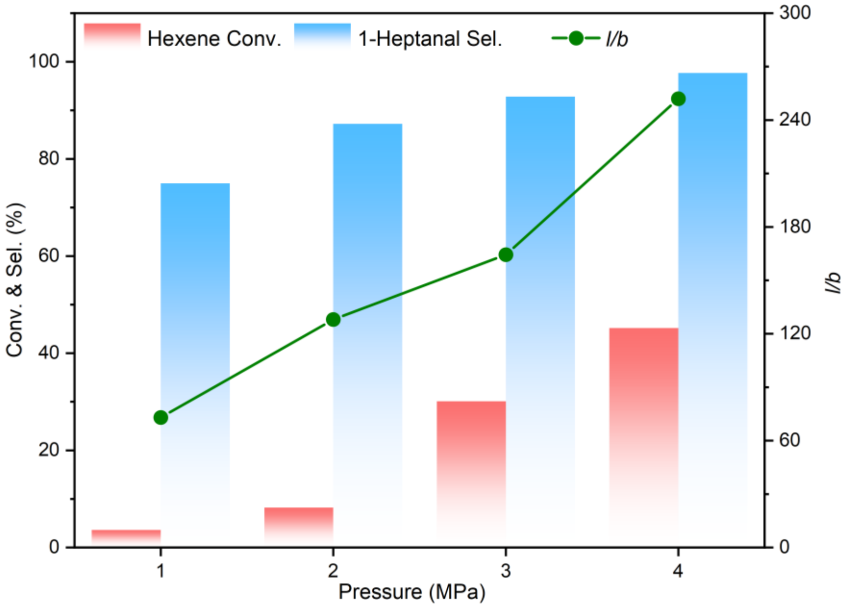

Figure S13. The optimization of the reaction pressure over the Rh@MEL-DMBT catalyzed hydroformylation. Reaction conditions: 20 mg of catalyst, 1 mmol hexene, 4.0 mL of toluene, 80°C, H_2/CO (1:1, v/v), 800 r.p.m., 1 h.

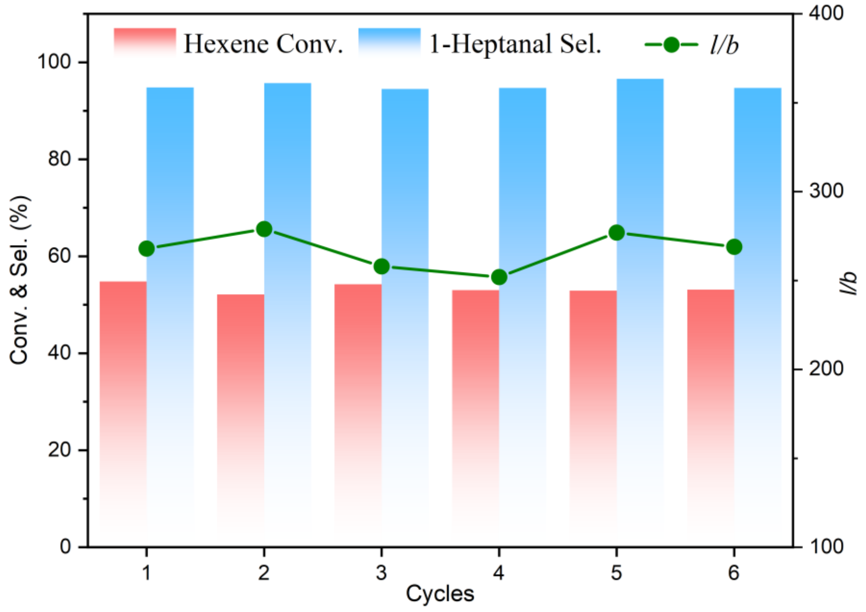

Figure S14. Stability test of Rh@MEL-DMBT catalyzed hydroformylation. Reaction conditions: 20 mg of catalyst, 1 mmol hexene, 4.0 mL of toluene, 80°C, 4 MPa of H_2/CO (1:1, v/v), 800 r.p.m., 1.5 h.

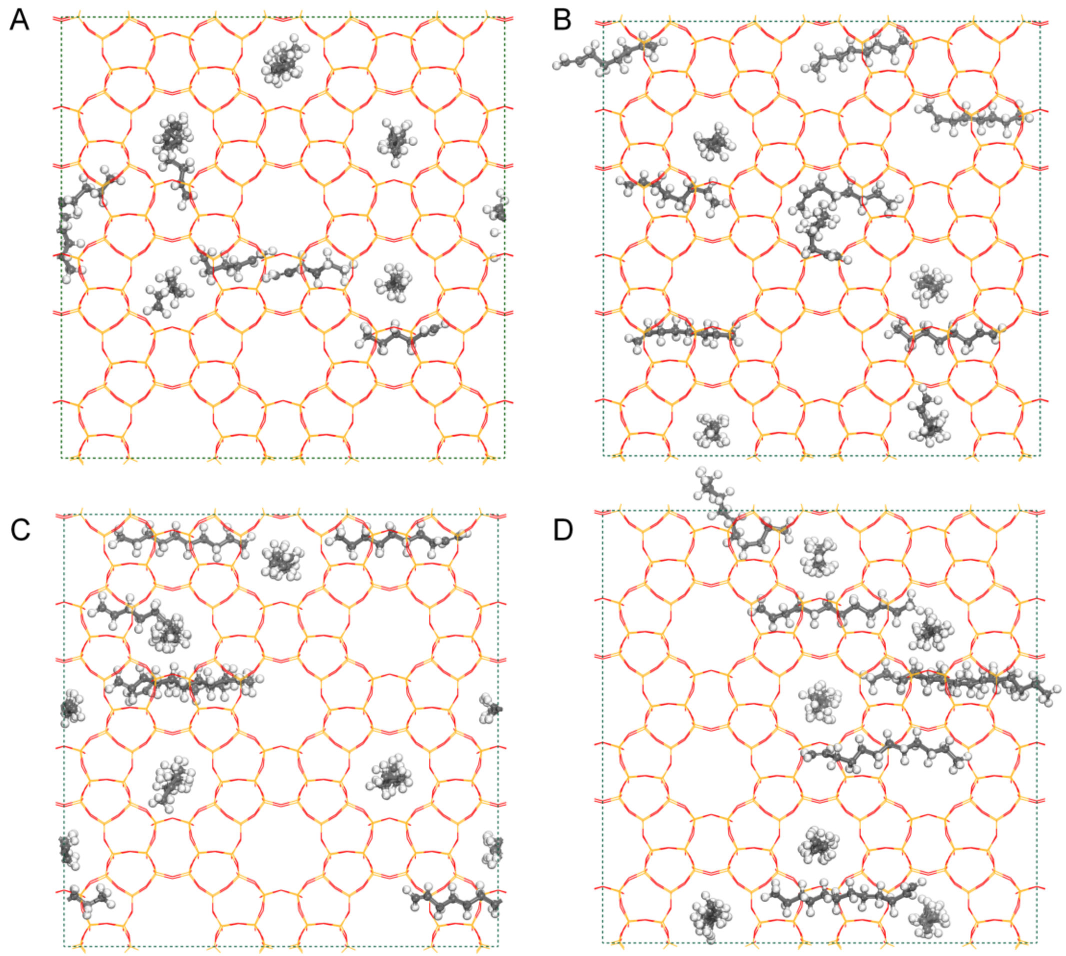

Figure S15. Initial structure of (A) C_6, (B) C_8, (C) C_{10} and (D) C_{12} olefin molecules adsorption in MEL zeolite.

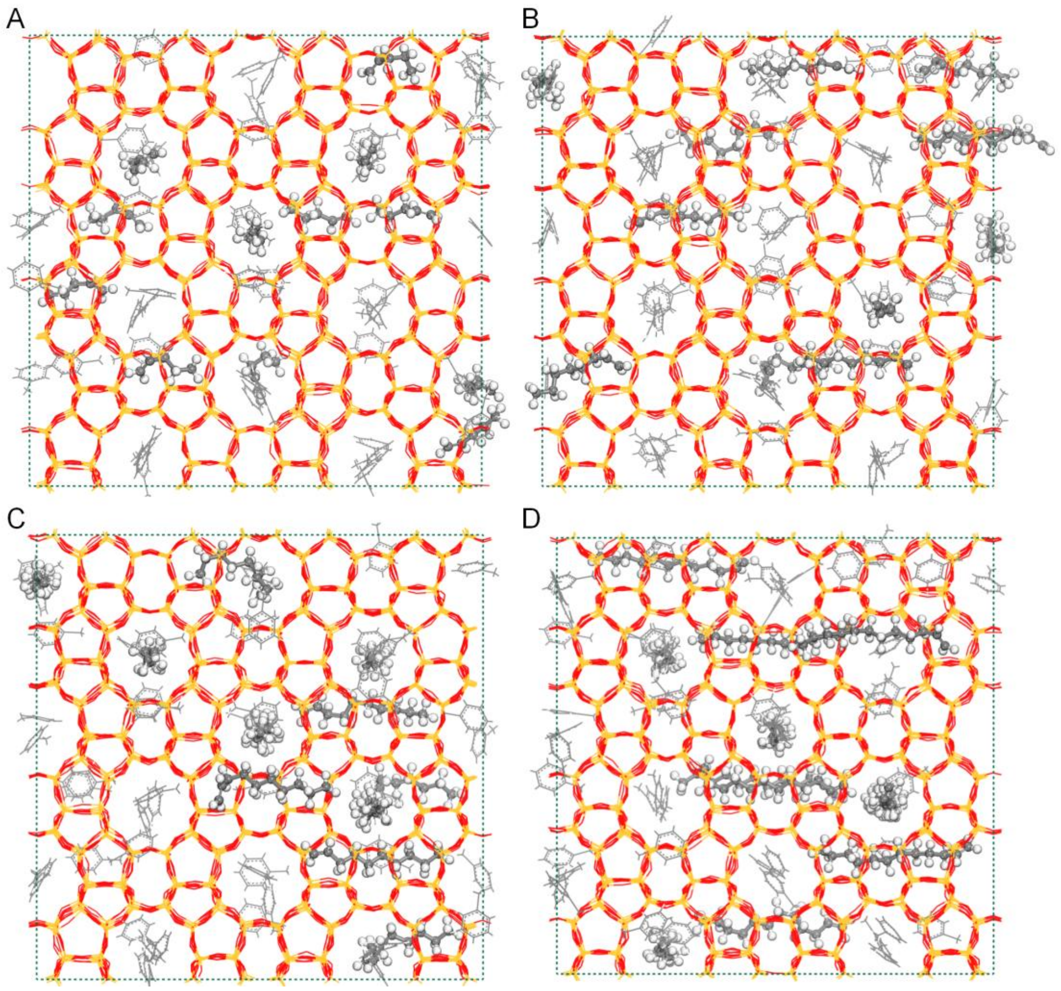

Figure S16. Initial structure of (A) C_6, (B) C_8, (C) C_{10} and (D) C_{12} olefin molecules, and toluene co-adsorption in MEL zeolite.

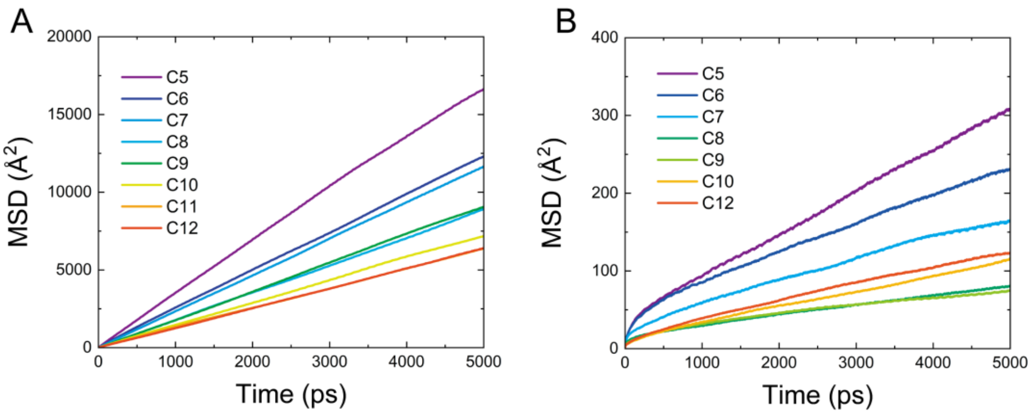

Figure S17. (A) Mean square displacement (MSD) of olefins with varying chain lengths in MEL zeolite. (B) In the presence of toluene, MSD of olefins with varying chain lengths in MEL zeolite.

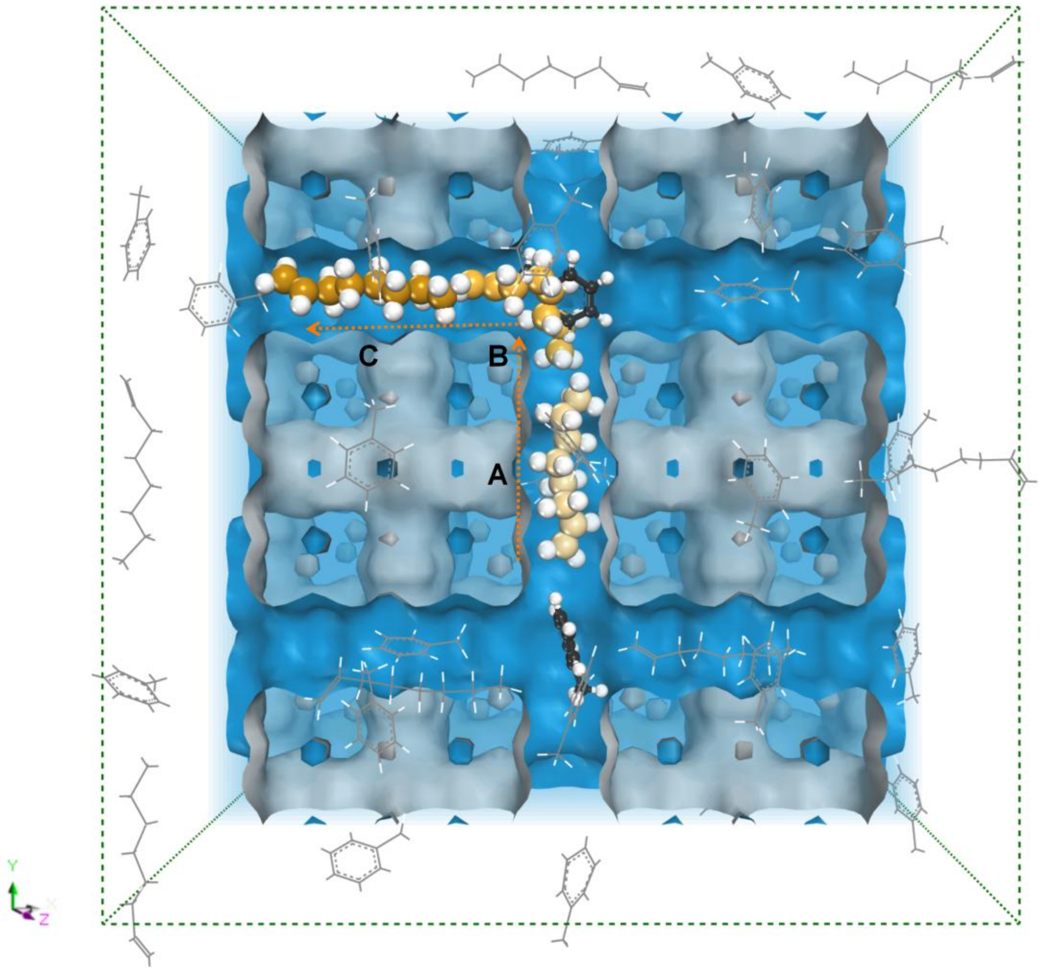

Figure S18. Schematic illustration of the diffusion pathway: Initially, the C_8 olefin (A, 8972 ps) is positioned along the Y-direction, while toluene occupies the channel intersection. The presence of toluene at the intersection blocks the diffusion of C_8. Subsequently, C_8 displaces the toluene molecule (B, 10002 ps) and diffuses along the X-direction (C, 10055 ps).

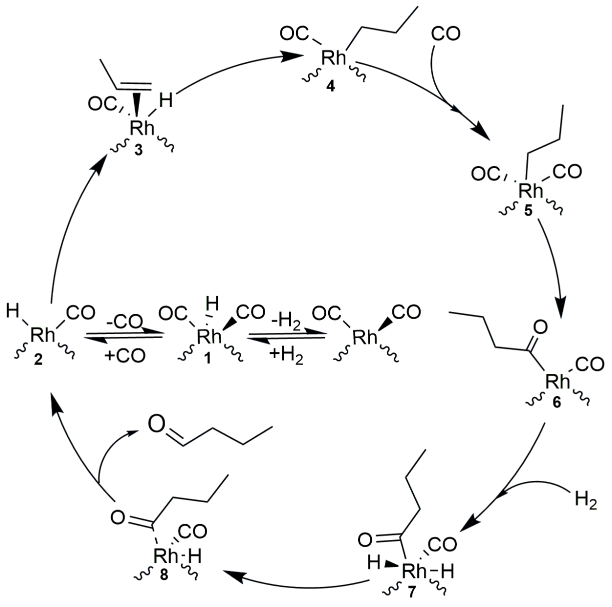

Figure S19. Illustrative diagram of the catalytic reaction pathway for production of linear butanal in the hydroformylation of propene with Rh@MEL-DMBT.

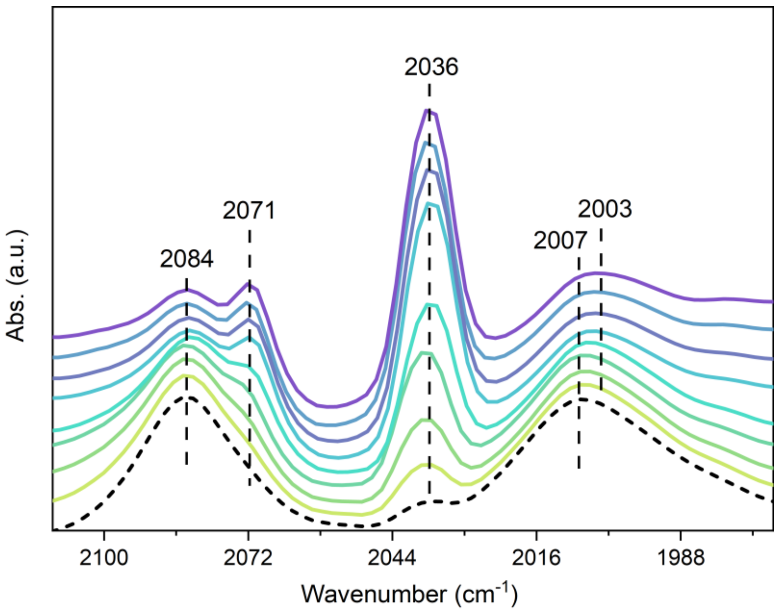

Figure S20. *In situ* IR spectra of propene hydroformylation over Rh@MEL-DMBT catalyst. Gaseous CO contributions were subtracted from the original spectra.[1]

Table S1. The Rh content of analysis in Rh@MEL catalysts.

Entry	Sample	Rh (wt%)[a]
1	Synthesized Rh@MEL	0.32
2	Spent Rh@MEL	0.32

[a]The elemental composition of rhodium in the synthesized and spent Rh@MEL catalyst was quantitatively analyzed by ICP-MS. Spent Rh@MEL refers to the sample after six cycles of reuse.

Table S2. The encapsulation efficiency was evaluated via 1-octene and cyclooctene hydrogenation over Rh@MEL catalysts.

Entry	Sample	TOF / hours^{-1} (1-octene)	TOF/ hours^{-1} (cyclooctene)	1-χ_{SiO_2}/χ_{MEL}[a]
1	Rh@MEL	18810	2620	0.94
2	Rh/SiO_2	25633	61539	-

[a]The parameter χ_{sample} ($TOF_{1\text{-}octene}/TOF_{cyclooctene}$) quantifies Rh encapsulation efficiency within Rh@zeolite, where 1-χ_{SiO_2}/χ_{MEL} represents the fraction of Rh located in zeolite channels. The reference value χ_{SiO2}, determined using SiO_2-supported Rh, accounts for intrinsic reactivity differences between substrates.

Table S3. Rh K-edge EXAFS Fitting Parameters[a] for Rh@MEL.

Path	CN	R (Å)	σ^2 (Å^2)	ΔE (eV)	R factor
Rh-Rh	1.2 ±0.8	2.72 ±0.02	0.006 ±0.005	2.2 ±2.2	0.018
Rh-O	3.6 ±0.5	2.05 ±0.02	0.005 ±0.002		

[a]S_0^2 was fixed as 0.75. Data ranges: $3.0 \leq k \leq 11.0$ Å^{-1}, $1.0 \leq R \leq 3.0$ Å . The scattering path for Rh-Rh is extracted from crystal structure of Rh metal, and the scattering paths for Rh-O are extracted from the Rh_2O_3.

Table S4. Catalytic performance of the Rh@MEL-DMBT catalyst in the hydroformylation of internal olefins.[a]

Entry	Substrate	Olefins conversion (%)	Selectivity for aldehydes (%)	*l*/*b*	Selectivity for isomerized alkenes (%)	Other products
1	2-hexene	3.8	77.2	33	26.3	<1
2	3-hexene	~0.2	0	/	100	0
3	2-octene	2.8	75.6	23.8	27	<1

[a]Reaction conditions: 20 mg of catalyst 80°C, 5 h, H_2 (2 MPa), CO (2 MPa), internal olefins (1 mmol), 4 ml of toluene. The *l*/*b* ratio represents the molar ratio of linear aldehyde to branched aldehyde in the product mixture.

Table S5. Optimizing DMBT dosage in the hydroformylation of 1-hexene over Rh@MEL-DMBT catalyst[a].

DMBT/Rh ratio (mol/mol)[b]	1-Hexene conv. (%)	1-Heptanal sel. (%)	2-Methylhexanal sel. (%)	Internal hexenes sel. (%)	*l*/*b*
0	69.6	69.9	12.4	16.9	5.6
1	56.8	83.1	4.8	11.5	17.3
2	52.9	87.1	2.6	11.2	32.5
3	49.9	90.8	1.6	8.3	56.7
4	46.6	94.2	0.8	4.5	118
6	43.8	96.3	0.25	2.8	385
8	43.7	96.3	0.25	2.8	385
40	43.5	96.1	0.25	3.1	384

[a]Reaction conditions: 20 mg of catalyst, 1-hexene (1 mmol), 80°C, 1 h, H_2 (2 MPa), CO (2 MPa), 4 ml of toluene.

[b]The molar amount of Rh refers to the total moles of rhodium present in the catalyst

Table S6. Time-dependent 1-hexene conversion and products selectivity over Rh@MEL-DMBT[a].

Reaction time (min)	1-Hexene conv. (%)	1-Heptanal sel. (%)	2-Methylhexanal sel. (%)	Internal hexenes sel. (%)	*l*/*b*
10	10.9	95.6	0.17	2.6	562
20	20.2	96.5	0.2	2.3	482
30	25.7	96.3	0.22	2.5	437
60	43.9	96.2	0.25	2.5	384
90	53.9	94.0	0.31	4.7	303
120	72.0	94.4	0.33	4.2	286
150	84.5	94.1	0.33	4.5	285
180	94.9	93.5	0.38	5.1	246
240	100	93.2	0.4	5.4	233

[a]Reaction conditions: 20 mg of catalyst, 1-hexene (1 mmol), 2 mg DMBT, 80°C, H_2 (2 MPa), CO (2 MPa), 4 ml of toluene.

Table S7. Time-dependent 1-hexene conversion and products selectivity over Rh/SiO_2[a].

Reaction time (min)	1-Hexene conv. (%)	1-Heptanal sel. (%)	2-Methylhexanal sel. (%)	2-Ethylpentanal sel. (%)	Internal hexenes sel. (%)	*l*/*b*[b]
30	19.0	41.1	12.0	0	45.9	3.5
60	31.1	35.7	11.3	0	52.1	3.2
90	39.4	30.6	10.2	0	60.5	3.0
120	74.6	32.8	11.8	0	55.8	2.8
180	89.3	42.4	17.7	0	38.8	2.4
240	99.6	43.5	24.2	9.7	23.3	1.3
300	99.8	44.4	35.1	11.1	9.1	1.0
360	99.6	47.0	39.3	11.9	1.1	0.9
420	99.5	46.8	40.8	12.1	0.1	0.9

[a]Reaction conditions: 20 mg of catalyst, 1-hexene (1 mmol), 80°C, H_2 (2 MPa), CO (2 MPa), 4 ml of toluene.

[b]The *l*/*b* ratio represents the molar ratio of linear aldehyde to the sum of all branched aldehydes in the product mixture.

Table S8. The conversion and selectivity results for the hydroformylation of different linear α-olefins over Rh@MEL-DMBT[a].

Substrates	Conv. (%)	Aldehydes sel. (%)	*l*/*b*	Internal alkenes sel. (%)	Other products (%)
1-Pentene (C_5)	99.2	96.7	318	2.3	<1
1-Hexene (C_6)	99.1	95.7	215	4.8	<1
1-Heptene (C_7)	80.4	85.5	86	14.1	<1
1-Octene (C_8)	92.1	86.3	152	15.8	<1
1-Nonene (C_9)	99.2	95.7	564	4.9	<1
1-Decene (C_{10})	99.3	96.7	601	2.8	<1
1-Undecene (C_{11})	99.1	95.1	477	4.2	<1
1-Dodecene (C_{12})	33.8	91.4	313	6.0	<1

[a]Reaction conditions: 20 mg of catalyst, α-olefins (1 mmol), 2 mg DMBT, 80 ℃, 5 h, H_2 (2 MPa), CO (2 MPa), 4 ml of toluene.

Table S9. Initial hydroformylation rates and regioselectivity of linear α-olefins over Rh@MEL-DMBT[a].

Entry	Substrates	TOF[b] (%)	*l*/*b*
1	1-Pentene (C_5)	1126	400
2	1-Hexene (C_6)	783	370
3	1-Heptene (C_7)	367	161
4	1-Octene (C_8)	877	193
5	1-Nonene (C_9)	1593	566
6	1-Decene (C_{10})	1922	613
7	1-Undecene (C_{11})	1131	418
8	1-Dodecene (C_{12})	418	267

[a]Reaction conditions: 20 mg of catalyst, α-olefins (1 mmol), 2 mg DMBT, 80°C, H_2 (2 MPa), CO (2 MPa), 4 ml of toluene.

[b]For the TOF measurements with different olefin substrates, the reaction time was adjusted to maintain the conversion at approximately 10%.

Table S10. The performance of the Rh@MEL-DMBT catalyst in a 10-fold scale-up test.

Olefin	Olefin conv. (%)	Aldehyde sel. (%)	*l*/*b*	Internal alkenes sel. (%)	Other products (%)
1-Hexene (C_6)	97.8	96.7	283	4.8	<1
1-Nonene (C_9)	98.3	97.2	539	3.5	<1
1-Decene (C_{10})	98.5	97.9	536	3.6	<1
1-Undecene (C_{11})	97.7	96.0	402	4.5	<1

[a]Reaction conditions: 200 mg of catalyst, α-olefins (10 mmol), 20 mg DMBT, 80°C, 5 h, H_2 (2 MPa), CO (2 MPa), 40 ml of toluene.

Table S11. The effect of stirring speed on the conversion and selectivity in 1-hexene hydroformylation catalyzed by Rh@MEL-DMBT[a].

Entry	Stirring speed (r.p.m.)	1-Hexene conv. (%)	Aldehyde sel. (%)	*l/b*	Internal alkenes sel. (%)
1	0	15.2	90.8	145	8.5
2	200	24.8	92.5	364	6.4
3	400	37.4	93.4	301	7.1
4	600	42.5	95.2	357	4.3
5	800	43.7	96.1	382	3.2
6	1000	44.1	96.6	329	3.7

[a]Reaction conditions: 20 mg of catalyst, 1-hexene (1 mmol), 2 mg DMBT, 80°C, H_2 (2 MPa), CO (2 MPa), 4 ml of toluene, 1 h.

Table S12. Fitted kinetic parameters for propene hydroformylation over Rh@MEL-DMBT catalysts derived from Langmuir-Hinshelwood modeling [a].

Entry	Pressure (MPa)	*k'*	*K'*	R^2	r_0
1	3.0	1.38	1.61	0.978	0.161
2	4.0	2.73	1.94	0.986	0.247

[a] Reaction conditions: 20 mg of catalyst, 4 mL of toluene, 80 ℃, CO/H_2 = 1:1. The kinetic parameters k' and K' were obtained from global fitting of the experimental data to the Langmuir-Hinshelwood modeling. The coefficient of determination (R^2) quantifies the proportion of variance in the dependent variable that is explained by the regression model. r_0 represents the theoretically predicted initial reaction rate derived from the kinetic model.

Supplemental Note 1: The derivation process of the dynamic equation

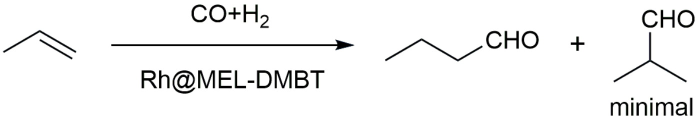

For the Rh@MEL catalytic system, the fundamental reaction steps have been streamlined into the subsequent seven stages. All elementary steps preceding the rate-determining step (RDS) were assumed to be in quasi-equilibrium, as per the equilibrium approximation. The carbonyl insertion step of the alkene (5) was identified as the rate determining step (RDS), as supported by DFT theoretical calculations, kinetic experiments, and infrared spectroscopy observations. All kinetic models were developed based on the Langmuirian assumption of homogeneous catalytic sites with equivalent reactivity.[10]

$$H_2 + 2M^* \rightleftharpoons 2H{\bullet}M^* \quad 1$$

$$CO + M^* \rightleftharpoons CO{\bullet}M^* \quad 2$$

$$C_3H_6 + M^* \rightleftharpoons C_3H_6{\bullet}M^* \quad 3$$

$$C_3H_6{\bullet}M^* + H{\bullet}M^* \rightleftharpoons C_3H_7{\bullet}M^* + M^* \quad 4$$

$$C_3H_7{\bullet}M^* + CO{\bullet}M^* \rightarrow C_3H_7CO{\bullet}M^* + M^* \quad 5$$

$$C_3H_7CO{\bullet}M^* + H{\bullet}M^* \rightleftharpoons C_3H_7CHO{\bullet}M^* + M^* \quad 6$$

$$C_3H_7CHO{\bullet}M^* \rightleftharpoons C_3H_7CHO + M^* \quad 7$$

$$[H_2] \times {\theta_{M*}}^2 \times K_{ad1} = {\theta_{H\cdot M*}}^2 \quad (1)$$

$$[CO] \times \theta_{M*} \times K_{ad2} = \theta_{CO\cdot M*} \quad (2)$$

$$[C_3H_6] \times \theta_{M*} \times K_{ad3} = \theta_{C_3H_6\cdot M*} \quad (3)$$

$$\theta_{C_3H_6\cdot M*} \times \theta_{H\cdot M*} \times K_1 = \theta_{\mathrm{C_3H_7}\cdot M*} \times \theta_{M*} \quad (4)$$

$$r = k\theta_{\mathrm{C_3H_7}\cdot M*} \times \theta_{CO\cdot M*} \quad (5)$$

$$\theta_{C_3H_7CO\cdot M*} \times \theta_{H\cdot M*} \times K_2 = \theta_{C_3H_7CHO\cdot M*} \times \theta_{M*} \quad (6)$$

$$\theta_{C_3H_7CHO\cdot M*} = K_{ad4} \times [C_3H_7CHO] \times \theta_{M*} \quad (7)$$

$$\theta_{M*} + \theta_{H\cdot M*} + \theta_{CO\cdot M*} + \theta_{C_6H_{12}\cdot M*} + \theta_{C_6H_{13}\cdot M*} + \theta_{C_6H_{13}CO\cdot M*} + \theta_{C_6H_{13}CHO\cdot M*} = 1 \quad (8)$$

Due to the large excess of reactants relative to the catalyst, the contribution of adsorbed intermediates to the bulk concentration was negligible. The solubility of propylene in toluene is assumed to follow Henry's law, where the dissolved gas concentration ($[C_3H_6]$) is proportional to its partial pressure ($p_{[C_3H_6]}$) at constant temperature, as expressed by eq 9

$$p_{[C_3H_6]} = k_{c,[C_3H_6]} \times [C_3H_6] \quad (9)$$

The gas-phase propylene is modeled as an ideal gas, following the eq 10. Vs and V_l respectively represent the volume of the steel reactor and the volume of the solvent (toluene), which remain constant throughout the reaction process. The concentration of propylene fully dissolved in toluene ($[C_3H_6]_{all}$) at any given reaction time is expressed as eq 11. $[C_3H_6]_p$ denotes the equilibrium concentration of propylene dissolved in toluene upon complete dissolution of residual gas-phase propylene, as calculable from eq 12. The final expression for $[C_3H_6]_{all}$ is given by eq 15.

$$p_{[C_3H_6]} \times Vs = n_{C_3H_6} \times R \times T \quad (10)$$

$$[C_3H_6]_{all} = [C_3H_6] + [C_3H_6]_p \quad (11)$$

$$[C_3H_6]_p = \frac{k_{c,[C_3H_{16}]} \times Vs \times [C_3H_6]}{R \times T \times V_l} \quad (12)$$

$$[C_3H_6]_{all} = [C_3H_6] \times \left(1 + \frac{k_{c,[C_3H_6]} \times Vs}{R \times T \times V_l}\right) \quad (13)$$

$$i = 1 + \frac{k_{c,[C_3H_6]} \times Vs}{R \times T \times V_l} \quad (14)$$

$$[C_3H_6]_{all} = [C_3H_6] \times i \quad (15)$$

Thus, the bulk concentrations of substrates ($[C_3H_6]$) and products ($[C_3H_7CHO]$) could be directly calculated using eq 16.

$$[C_3H_6]_0 - [C_3H_6]_{all} = [C_3H_7CHO] \quad (16)$$

$$[C_3H_6]_0 - [C_3H_6] \times i = [C_3H_7CHO]$$

$$\theta_{M*} = \frac{1}{P+Q[C_3H_6]} \tag{17}$$

$$P = A + B \times K_{ad4} \times [C_3H_6]_0 \tag{18}$$

$$Q = C \times K_{ad3} - B \times K_{ad4} \times i \tag{19}$$

$$A = 1 + \sqrt{K_{ad1} \times [H_2]} + K_{ad2} \times [CO] \tag{20}$$

$$B = 1 + \frac{1}{K_2 \times \sqrt{K_{ad1} \times [H_2]}} \tag{21}$$

$$C = 1 + K_1\sqrt{K_{ad1} \times [H_2]} \tag{22}$$

The intermediate parameters P, Q, A, B, and C are defined by eq 18 and 19-22. Based on these definitions, the apparent reaction rate (*r*) was derived from eq 5 and expressed as eq 23. Throughout the analysis, the concentrations of H_2 and CO were assumed to remain constant, as the pressure change during the reaction was negligible.

$$r = \frac{k\prime \times [C_3H_6]}{(1+K\prime \times [C_3H_6])^2} \tag{23}$$

$$k' = \frac{R}{P^2} \tag{24}$$

$$K' = \frac{Q}{P} \tag{25}$$

$$R = k \times K_1\sqrt{K_{ad1} \times [H_2]} \times K_{ad2} \times [CO] \times K_{ad3} \tag{26}$$

The parameter X is defined as the conversion, represented by eq 27. Finally, the relationship between the conversion X and t can be expressed by eq 28.

$$X = \frac{[C_3H_6]_0\text{-}[C_3H_6] \times i}{[C_3H_6]_0} \tag{27}$$

$$t - \gamma = \frac{1}{k\prime}\left(-\ln(1-X) + \left(2K'\frac{[C_3H_6]_0}{i} + K'^2\frac{[C_3H_6]_0{}^2}{i^2}\right)X - \frac{1}{2}K'^2\frac{[C_3H_6]_0{}^2}{i^2}X^2\right) \tag{28}$$

The concentrations of H_2 ($[H_2]$) and CO ([CO]) in the liquid phase were determined by their respective gas partial pressures through Henry's law. Based on reported solubility and adsorption constants for H_2 and CO in analogous systems, we established that the conditions $K_{ad1}[H_2] \ll 1$ and K_{ad2} $[CO] \ll 1$ were satisfied, where K_{ad1} and K_{ad2} represent the adsorption equilibrium constants for

H_2 and CO, respectively. Consequently, the initial catalytic reaction rate (r_0) may be reduced to eq 29. Under the condition of maintaining constant propylene partial pressure and a fixed CO/H_2 ratio of 1, we could infer that [H_2] and [CO] were proportional to the total pressure (p), according to the Henry's law. Therefore, r_0 was proportional to $p^{1.5}$ when the RDS was Step 5.

$$r_0 = \frac{\mathrm{R}[C_3H_6]}{\left(1 + BK_{ad4}[C_3H_6]_0 + K_{ad3}[C_3H_6] - BK_{ad4}[C_3H_6]\right)^2}$$

$$\approx \frac{\mathrm{R}[C_3H_6]}{\left(1 + BK_{ad4}[C_3H_6]_0 + K_{ad3}[C_3H_6] - BK_{ad4}[C_3H_6]\right)^2}$$

$$= \frac{kK_1\sqrt{K_{ad1}\times[H_2]}\times K_{ad2}\times[CO]\times K_{ad3}\times\ [C_3H_6]}{\left(1+BK_{ad4}[C_3H_6]_0+K_{ad3}[C_3H_6]-BK_{ad4}[C_3H_6]\right)^2} \quad (29)$$

$$r_0 \propto\ p^{1.5} \quad (30)$$

The constant terms in the two-parameter model (Eq. 28) were determined through nonlinear regression of experimental data at the designated pressure. These parameters were then utilized in Eq. 30 to calculate the corresponding initial reaction rates (r_0). Subsequent linear regression analysis of the pressure dependence of r_0 confirmed carbonyl insertion as the rate-determining step in the catalytic cycle.

References

1. Zhang X, Yan T, Hou H *et al.* Regioselective hydroformylation of propene catalysed by rhodium-zeolite. *Nature* 2024; **629**: 597–602.
2. Cho HJ, Kim D, Li J *et al.* Zeolite-encapsulated Pt nanoparticles for tandem catalysis. *J Am Chem Soc* 2018; **140**: 13514–20.
3. Sun H. COMPASS: an ab initio force-field optimized for condensed-phase applications overview with details on alkane and benzene compounds. *J Phys Chem B* 1998; **102**: 7338–64.
4. Deore S, Simoncic P and Navrotsky A. Molecular mechanics studies of thionin blue in zeolite mordenite. *Microporous Mesoporous Mater* 2008; **109**; 342–9.
5. Liu Z, Kan X, Gao M *et al*. Asymmetric rotations slow down diffusion under confinement. *Nat Commun* 2025; **16**: 2018.
6. Li Q, Das S, Zhang Y *et al.* Ethene hydroformylation catalyzed by rhodium dispersed with zinc or cobalt in silanol nests of dealuminated zeolite beta. *J Am Chem Soc* 2023; **145**: 2911–29.
7. Ro I, Qi J, Lee S *et al.* Bifunctional hydroformylation on heterogeneous Rh-WO_x pair site catalysts. *Nature* 2022; **609**: 287–92.
8. Yu Z, Zhang S, Zhang L *et al.* Suppressing metal leaching and sintering in hydroformylation reaction by modulating the coordination of Rh single atoms with reactants. *J Am Chem Soc* 2024; **146**: 11955–67.
9. Liu Y, Liu Z, Hui Y *et al.* Rhodium nanoparticles supported on silanol-rich zeolites beyond the homogeneous Wilkinson’s catalyst for hydroformylation of olefins. *Nat Commun* 2023; **14**: 2531.
10. Liu B, Huang N, Wang Y *et al*. Insights into the activity screening and hydroformylation kinetics of Rh-based bimetallic phosphides. *ACS Catal* 2021; **11**: 15235–43.
